# Quantitative Understanding of the Decision-Making Process for Farm Biosecurity Among Japanese Livestock Farmers Using the KAP-Capacity Framework

**DOI:** 10.3389/fvets.2020.00614

**Published:** 2020-09-11

**Authors:** Kohei Makita, Elly Steenbergen, Lisa Haruta, Saddam Hossain, Yuki Nakahara, Yuto Tamura, Takuto Watanabe, Hazumu Kadowaki, Shingo Asakura

**Affiliations:** ^1^Veterinary Epidemiology Unit, School of Veterinary Medicine, Rakuno Gakuen University, Ebetsu, Japan; ^2^Quantitative Veterinary Epidemiology Group, Wageningen University & Research, Wageningen, Netherlands; ^3^Department of Medicine and Surgery, Faculty of Veterinary Medicine, Chittagong Veterinary and Animal Sciences University, Chittagong, Bangladesh

**Keywords:** KAP analysis, capacity, biosecurity, decision-making, structural equation modeling

## Abstract

In a globalized world, the frequency of transboundary livestock infectious diseases is increasing, and strengthening of farm biosecurity is vital to stabilize food production. The aim of this study was to understand the decision-making process for farm biosecurity among Japanese livestock farmers. Postal surveys using structured questionnaires were conducted on beef, dairy, pig, and layer farms in Hokkaido and Saitama Prefectures, which represent the principal production area and peri-urban Tokyo, respectively, as well as randomly selected broiler farms across Japan. The question items included the attributes of farms and owners, disease experiences, related associations and sources of hygiene information, attitude toward hygiene management, and compliance with the Standards of Rearing Hygiene Management (SRHM). The compliance rates were compared between livestock sectors. Univariable analyses were conducted using combined data from both prefectures, with the compliance rate as the outcome variable and the questionnaire items as explanatory variables, in generalized linear models. Exploratory factor analyses were conducted using the variables with *p* < 0.2 in the univariable analyses. The factors identified were classified into knowledge, attitude, capacity, practice, and structural equation modeling (SEM) was performed. The questionnaires were completed and returned by 97 and 66 beef cattle, 86 and 136 dairy, 67 and 45 pig, 20 and 39 layer farmers in Hokkaido and Saitama Prefectures, respectively, and 95 broiler farms. The compliance rate was significantly higher among broiler farms (88.9%) compared with the other sectors, followed by pig (77.1%), layer (67.2%), dairy (63.8%), and beef (59.1%) farms in Hokkaido Prefecture, and layer (64.9%), pig (60.0%), dairy (58.5%), and beef (57.6%) farms in Saitama Prefecture. Based on SEM, the decision-making process from greater knowledge to higher attitude, and from higher attitude to greater compliance with the SRHM were significant (*p* < 0.01) in all sectors. Higher capacity was significantly associated with higher knowledge in dairy, pig,break and layer farms (*p* < 0.01), and with higher compliance in beef, pig, and layer farms (*p* < 0.05). These results suggest that the provision of targeted hygiene knowledge to livestock farmers and the support to smallholder farms would improve biosecurity through elevated attitudes and self-efficacy.

## Introduction

Infectious diseases remain a significant threat to the livestock industry in a globalized world. For example, African swine fever rapidly expanded from its original territory in sub-Saharan Africa to Georgia in 2007 (1), with subsequent outbreaks in the Caucasus, eastern and central Europe, and the Baltic countries (2, 3), as well as wide regions of east and southeast Asian countries, since 2018 (4, 5). Moreover, increased travel and trade, which also increases the chance of illegal importation of infected livestock products, pose an elevated risk of long-distance international transmission of infectious agents (6). Infectious animal disease epidemics not only cause severe economic damage, but also affect the mental well-being of farmers, veterinarians, and civilians in affected areas, as seen worldwide in the foot-and-mouth disease (FMD) epidemic (7–9).

Farm biosecurity is an integral part of livestock production (10). It prevents the introduction of infectious agents into farms thereby reducing the economic burden caused by infectious disease outbreaks in animals. In 2004, the Ministry of Agriculture, Forestry and Fisheries (MAFF) of Japan established the Standards of Rearing Hygiene Management (SRHM), specified by the Act on Domestic Animal Infectious Diseases Control in Japanese law (11, 12). The SRHM incorporated 10 basic on-farm standards for cattle, pig, and poultry farms as minimum hygiene standards, including hygienic rearing, disinfection at farm entrances and vehicles, quarantine upon animal introduction, and the acquisition of knowledge regarding infectious disease prevention. However, in 2010, major outbreaks of FMD and highly pathogenic avian influenza (HPAI) occurred in Japan, during which a total of 1.8 million chickens, 230,000 pigs, and 70,000 cattle were culled, causing serious losses to the livestock sectors (13, 14). After these major outbreaks, in 2011, the MAFF revised the SRHM by setting species-specific standards for (1) cattle, buffaloes, deer, sheep, and goats, (2) pigs and domesticated wild boars, (3) poultry, and (4) horses, and by increasing the number of hygiene standards from 10 to 22–25 items (15). Hereby, more emphasis was placed on the establishment of hygienic zones, farm entrance restrictions, the securing of land for burying carcasses after emergency culling, and the early detection and reporting of infectious diseases (12). Although substantial efforts were made to implement the SRHM on livestock farms, in 2013, porcine epidemic diarrhea (PED), which was causing a global pandemic, occurred in Japan, resulting in the deaths of over 500,000 pigs in the country (16). Reflecting on the findings of epidemiological investigations on PED (16) and critical discussions by experts (12), the SRHM were revised to include the prevention of contact between animal carcasses and wildlife, the establishment of a minimum temperature to heat human food waste for animal feeding, and the avoidance of leakage during the transportation of carcasses and animal excrement (17). The SRHM provided clear guidance to Japanese livestock farmers regarding biosecurity; however, compliance remains a challenge (18). The recent incursion of classical swine fever into the wild boar population has been causing infections on Japanese pig farms since 2018 (19, 20), and thus, improvements in farm biosecurity are becoming increasingly important.

Several frameworks have been proposed to understand the decision-making process in health-related practices. For example, the Health Belief Model (HBM), which consists of four components—perceived susceptibility to a health threat, severity of the threat, benefits to prevent the threat, and barriers to the preventive behavior (21)—has been reported in a review of 24 studies to affect preventive actions in the order of barriers, susceptibility, benefits, and severity (22). The Protection Motivation Theory (PMT) proposes that two cognitive processes—threat appraisal and coping appraisal—determine the conduct or inhibition of protective actions (23). Threat appraisal process evaluates the factors that increase or decrease the probability of making the maladaptive responses such as smoking or not wearing a seat-belt. Intrinsic (e.g., bodily pleasure) and extrinsic rewards (e.g., social approval) increase the probability of the maladaptive response, while assessed severity of the threat and perceived vulnerability to the threat reduce the probability. Coping appraisal is increased by judgments about the efficacy of a preventive response and one's ability to adapt the response successfully, and is decreased by the response cost (23). PMT has been applied in animal health to determine the factors associated with biosecurity practices during an equine influenza outbreak in Australia (24) and with the perception of vulnerability to future outbreaks (25) using logistic regression analysis. The Theory of Reasoned Action (TRA) states that an individual's behavior may be predicted by the strength of the intention, which depends on a combination of attitudes and subjective norms (26, 27). The Theory of Planned Behavior (TPB) is an extension of the TRA. In the TPB, in addition to attitudes and subjective norms, “perceived behavioral control (PBC),” which accounts for the belief in self-efficacy and perceived difficulties, is assumed to influence behavioral intentions (28). PBC not only affects behavioral intentions, but is also directly related to actual behaviors (27, 28). The TRA and TPB are applied in qualitative studies on the decision-making process of farm disease control (27–29). Another framework to identify knowledge gaps, cultural beliefs, or behavioral patterns that may be obstacles to the control of infectious diseases is the Knowledge, Attitudes, and Practice (KAP) framework (30). Numerous qualitative studies on the KAP framework have been carried out in animal health worldwide, and some studies, for example, on rabies, have applied this framework using uni- and multivariable linear analyses (31). The limitation of this approach is that linear analysis measures direct and indirect associations between factors and key disease preventive practice(s), but cannot infer complex mechanisms in the decision process. Toma et al. (10) modeled flows from several attitude nodes to the biosecurity behaviors of British cattle and sheep farmers, and Kadowaki et al. presented the flow of sociological factors to the KAP framework as a sequence (better livelihood enhances knowledge, higher knowledge leads to better attitudes, and better attitudes initiate good practices) in voluntary rabies control measures in Vietnam (32) using structural equation modeling (SEM). SEM is not intended to discover causes (33), but rather, to assess the soundness of causal relationships formulated a priori (10). SEM is useful for understanding decision-making mechanisms because it can distinguish latent variables (e.g., KAP) from observed variables (34) and assess the relationships between latent variables using observed variables.

The initial purpose of this study was to gain a better understanding of the influence of socioeconomic factors in the practice of biosecurity measures on livestock farms in Japan in order to further improve the level of biosecurity. During focus group discussions (FGDs) and an analysis of results from a postal survey using linear models, in addition to the KAP framework, the importance of the capacity of farmers, such as the size of the operation and the age of owners in family-owned farms, was perceived. A preliminary analysis on pig farmers, using the same data with the present study, is published in Japanese (35). Therefore, this study assesses the decision-making mechanism of farm owners in regard to biosecurity based on the KAP plus capacity (KAP-Capacity) framework.

## Materials and Methods

### Study Areas

**Figure 1:**
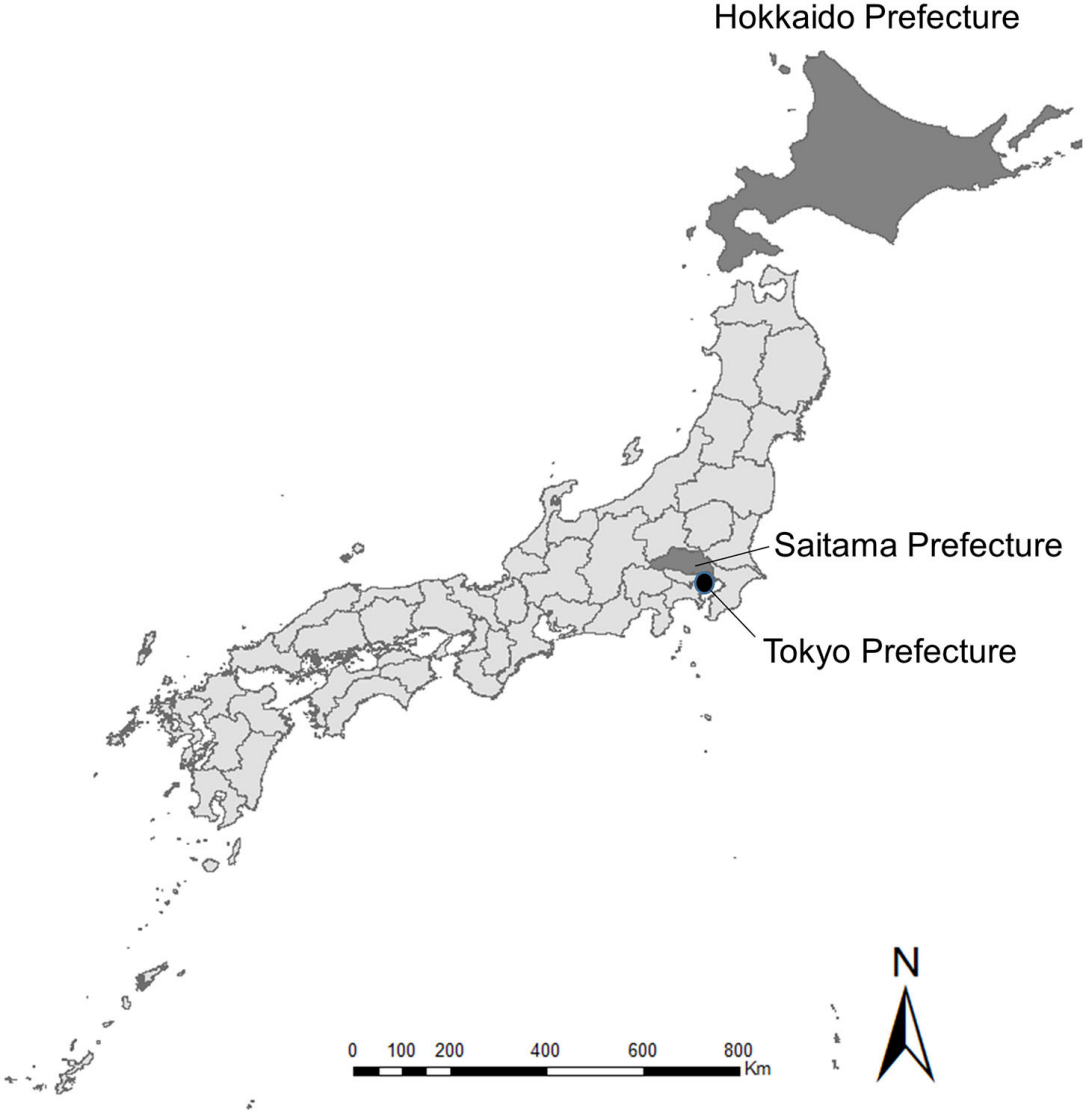
A map of Japan indicating the locations of Hokkaido, Saitama, and Tokyo Prefectures.

Studies on beef and dairy cattle, pig, and layer farm owners were conducted in Hokkaido and Saitama Prefectures, Japan, to compare livestock production (Hokkaido) and peri-urban areas (Saitama) (Figure 1). In Japan, broiler producers are no longer considered farmers, but rather, companies, so a study on these companies was conducted at the national level.

### Focus Group Discussions

Between October 2013 and January 2014, at least one FDG was held for each of the livestock sectors, except for the broiler sector, to collect information that could be used to develop questionnaires. For the beef and dairy cattle, pig, and layer hen sectors, FDGs were held with people from both Hokkaido and Saitama Prefectures. For the broiler sector, semi-structured interviews were carried out with hygiene management veterinarians employed in broiler companies in Miyazaki and Hokkaido Prefectures. Each FGD, other than that for the broiler sector, was performed with a small group of people (5–6 persons) and included female farmers (or the wives of farm owners) and employees of livestock associations to gain different perspectives on the topics discussed. The discussions lasted a total of 80 min (two 40-min discussions with 10-min break in between), during which time, topics such as hygiene management, the influence of changes in the SRHM, livestock-related associations, and requirements for a questionnaire that could be easily interpreted and completed by farmers were discussed. All the FGDs were facilitated by an epidemiologist who was trained in participatory epidemiology and had 5-years' experience of the application in research.

### Postal Surveys

Based on the results of the FDGs and semi-structured interviews, livestock sector-specific questionnaires were constructed and then pretested on farmers and veterinarians. Table 1 shows a list of the question items with respect to farmer attributes, farm information, experience with animal diseases, cooperating associations, source of hygiene information, attitude toward hygiene information and management, and compliance with the SRHM. The questionnaire for the broiler sector was slightly different from that used in the other livestock sectors, as these are considered business companies owning several farms.

**Table 1:** Content of the questionnaire for farmers.

**Category**	**Questionnaire item**
Farmer's attributes	Age, gender, household size, schooling years, working time, and farming years of the farmer.
Farm information	Level of urbanization, year of farming since establishment, successor availability, number of livestock, housing facilities, management type, number of farm workers, whether the farm is adjacent to the main road, the density of neighboring farms, distance to the nearest farm, and the source of feed
Experience of diseases	Occurrence and impact of disease symptoms, a list of diseases experienced in last decade, and number of disease occurrences
Associations and information sources	Related associations, sources of hygiene information, and attendance of lectures and seminars about animal health
Attitudes toward hygiene information and management	Priority of information sources and activity, presence of change in attitude toward SRHM after its revision, and the reasons for this change, satisfaction level for hygiene management on the own farm and by the government, and the level of change in communication with other farmers after the revision of SRHM
Compliance with the Standards of Rearing Hygiene Management (SRHM)	Compliance with each standard described in the SRHM, categorized under five topics: the prevention of introduction with fomites, limitation of access to the farm, prevention of infection from wildlife, prevention of within-farm spread, and maintenance of preparedness

In Hokkaido Prefecture, questionnaires were sent to all beef cattle farmers belonging to Japan Agricultural Cooperatives (JA) in the Engaru and Yubetsu areas, those who attended technical workshops held in JA in the Tokachi area and the Hokkaido Dairy and Livestock Association (questionnaires were filled out at the workshops), all dairy farmers belonging to JA in the Engaru and Yubetsu areas, all pig farmers belonging to the Hokkaido Pig Farm Producers Association, and all layer farmers in Chitose, Ebetsu, Eniwa, and Ishikari cities, with assistance from the respective organizations. In Hokkaido, there were no integrated beef and dairy producers associations which can directly send questionnaires to farmers, and several intensive production areas were purposively selected. For layer farming, there were a few large scale companies, and there was no integrated farmers' association. Therefore, reachable cities from Rakuno Gakuen University were visited to enroll study participants. In Saitama Prefecture, questionnaires were sent to all beef and dairy cattle, pig, and layer farmers belonging to the Saitama Livestock Association, with assistance from the organization. For the broiler sector, sets of five questionnaires were sent to 50 randomly selected broiler-producing companies from a list published by the Japan Chicken Association, which has 81 member producers (36). The sample size for broiler companies was determined based on the available resources.

### SRHM Compliance Rates

The SRHM compliance rates were calculated as a proportion of SRHM items complied out of all the SRHM items for livestock species. Table 2 shows the SRHM items in the categories prepared following careful discussions by the authors: (1) prevention of disease incursion from fomites and animals, (2) limitation of access to a farm, (3) prevention of infection from wildlife, (4) prevention of within-farm spread, and (5) maintenance of preparedness. Some items such as provision of drinkable water and rearing animals with suitable density were associated with animal ethics and not necessarily with biosecurity; however they were classified into the most suitable categories. The SRHM included a few items specific to large-scale farms (adult cattle and buffalo: >300 heads; heifer, deer, sheep, and goats: >3,000 heads; poultry and quail: >100,000 birds; and ducks: >10,000 birds); however, for the purpose of analysis, in terms of compliance rate, these items for large-scale farms were excluded from the denominator to deal with all the respondents unweighted, regardless of farm size.

**Table 2:** Standards of rearing hygiene management.

**Category**	**Hygiene standards**
Prevention of disease incursion with fomites and animals	Disinfection of vehicles; Disinfection of hands and shoes of those who enter to the farm building; Provision of clothes and shoes only for hygiene control area (*only for pig and poultry farmers*); Cleaning or disinfection of materials directly used for animals when carry them in hygiene control area; Prohibition of carrying clothes and shoes used abroad into the farm; Quarantine of animals under segregation from other animals for certain period when introducing into the farm; Heat treatment of recycled feed (*only for pig farmers*)
Limitation of access to the farm	Segregation of hygiene control area from the other areas; Placement of a signboard indicating the hygiene control area; Limit of access for those who entered other farms or recently returned from abroad
Prevention of incursion from wildlife	Prevention of wildlife feces entering to feeding and water facilities; Provision of drinkable water; Placement of nets preventing entrance of wild birds (*only for poultry farmers*); Pest control, repair of damaged roof and walls (*only for poultry farmers*)
Prevention of within-farm spread	Change (disposal) or disinfection of materials to which body fluid of animals got attached, at each use; Cleaning and disinfection of a barn or cage after being emptied; Rearing animals with suitable density
Maintenance of preparedness	Collecting up-to-date information on prevention of animal infectious diseases; Immediate report of specific symptoms by law to the Livestock Hygiene Service Centre (LHSC) and restriction of animal movement; Immediate call of veterinarians when animals are sick without specific symptoms by law; Daily health check of animals; Removal of dirt and health check at selling out animals; Securing a land to bury culled animals; Record keeping for early identification of source of infection
Items for large scale farms (excluded from the analyses)	Designate one veterinarian or clinic, who tightly communicate with LHSM and regularly guide animal health management per farm, for all farms belonging to the company; Farm employees are aware that LHSC should be called without seeking permission of farm owner or manager immediately after the symptoms of specific diseases are detected

The compliance rates were compared among livestock species in Hokkaido and Saitama Prefectures separately using a generalized linear model (GLM) with quasi-binomial errors, as overdispersion was observed in the compliance rates. As broiler farms were sampled from the entire country, all broiler farm responses were included in the models for both prefectures. For dairy, beef, pig, and layer hen farmers, the compliance rates were compared between Hokkaido and Saitama Prefectures using GLMs.

### Comparisons of Farm Capacity and Density Between Hokkaido and Saitama Prefectures

To understand the differences in farm capacity and farm density between Hokkaido and Saitama Prefectures, capacity-related factors—the age of the owners, number of animals raised, number of farm workers, including the owner, the availability of successors, and the shortest distance to a farm raising the same species were compared between the two prefectures for beef and dairy cattle, pig, and layer farms. Capacity related factors were identified during above mentioned FGDs; as the owner gets older physical ability would be weak; large-scale farms with large numbers of animals and workers have greater work force; and availability of successor would be a good motivation of investments. The age of the owners was compared using *t*-tests when the data were normally-distributed, and the Wilcoxon rank sum test otherwise. The total numbers of animals in dairy and layer farms were compared using GLMs with quasi-Poisson errors. Beef cattle and pig farms were categorized according to management types (farrow-to-finisher, fattening, growing, and reproduction for beef cattle farms; and farrow-to-finisher, fattening, and reproduction for pig farms), and the total numbers of animals within these types were compared using the Wilcoxon rank sum test. The numbers of farm workers were compared using GLMs with quasi-Poisson errors. The proportions of farms with a successor were compared using chi-squared tests. The shortest distance to a farm raising the same species was also compared using the Wilcoxon rank sum test.

### Univariable Analysis of the Factors Affecting Compliance With the SRHM

We assumed that a decision making framework for implementing biosecurity measures would be common between Hokkaido and Saitama Prefectures, and as the sample sizes of the two prefectures were not so large, univariable analyses were conducted after combining both prefectures in terms of dairy and beef cattle, pigs, and layer farmers. For beef and dairy cattle, pigs, layer hen, and broiler producers, univariable analyses were conducted using GLMs with quasi-binomial errors, as overdispersion was commonly observed, selecting the compliance rate as the outcome variable and questionnaire items as the explanatory variables.

### Exploratory Factor Analysis

For each livestock species, factors with *p* < 0.2 were used for the exploratory factor analysis. When variables with similar socioeconomic meanings were identified by the investigators, those with the most direct meaning were selected to keep the number of variables as small as possible. For the practice of the SRHM, category-specific compliance rates were used for the analysis. According to our hypothesis, higher knowledge (K) would foster a better attitude (A), and a better attitude would determine the decision to conduct biosecurity practices (P). In addition, we hypothesized that higher capacity (Capacity) would facilitate gaining animal health knowledge, and that capacity would be needed to implement practices. Using parallel analysis scree plots plotted with the fa.parallel() function in the package “psych” (37) in R, the validity of the number of factors—four for the KAP-Capacity framework—was checked. Exploratory factor analyses specifying four factors were performed using the fa() function in the “psych” (37) and “GPArotation” packages in R (38), and the factors were checked based on the variables grouped together in terms of whether they were representative of K, A, P, and Capacity.

### Structural Equation Modeling

SEM was performed using the “lavaan” package in R (39). A path graph, which shows the structural part of the model, was designed selecting K, A, P, and Capacity as latent variables, and the remaining questionnaire variables from the exploratory factor analysis and category-specific SRHM compliance rates were used as the explanatory variables. To improve the model fit, in model tuning, non-significant variables in the exploratory factor analysis were either removed from the model or allocated to other factors based on the meanings of the variables. As the data sets included categorical and dichotomous variables and missing values, SEM was performed using mean-adjusted weighted least squares (WLSM) estimation, which is the robust version of weighted least squares (WLS) estimation (34). The fit indexes used were the significance of model chi-square (χ^2^), Tucker–Lewis Index (TLI), root mean square error of approximation (RMSEA), and standardized root mean square residual (SRMR). For a satisfactory fit, a model should have a non-significant model-χ^2^ (*p* > 0.05), TLI > 0.900, RMSEA < 0.08, and SRMR < 0.100 (40, 41). For all analyses, R statistical software version 3.6.1 (42) was used.

## Results

### Questionnaire Responses

The response rates for beef cattle, dairy, pig, and layer farmers in Hokkaido and Saitama Prefectures were 33.3% (97/291) and 41.5% (66/159), 29.7% (86/290) and 44.2% (136/308), 34.5% (67/194) and 36.0% (45/125), and 33.3% (20/60) and 39.8% (39/98), respectively. The response rate for broiler farms belonging to commercial companies was 38.0% (95/250).

### Compliance With the SRHM

Table 3 shows a comparison of SRHM compliance rates between livestock species in Hokkaido and Saitama Prefectures. In both prefectures, the mean compliance rates for beef cattle farms were the lowest among all livestock species (59.1 and 57.6% in Hokkaido and Saitama Prefectures, respectively). In Hokkaido Prefecture, the mean compliance rates of dairy (63.8%) and layer farms (67.2%) were not significantly different from that of beef cattle farms (*p* > 0.05), but the mean compliance rate of pig farms (77.1%) was significantly higher (*p* < 0.001). Conversely, in Saitama Prefecture, the mean compliance rate of layer farms (64.9%), but not those of dairy (58.5%) and pig farms (60.0%), was significantly higher than that of beef cattle farms (57.6%, *p* = 0.001). The compliance rate of broiler farms (88.9%) was significantly higher than any other species (the 95% confidence interval of the compliance rate did not overlap with any other species) in both prefectures.

**Table 3:** Comparison of the Standards of Rearing Hygiene Management compliance rates between livestock species in Hokkaido and Saitama Prefectures.

**Livestock species**	**Compliance rate (%) (95% CI)**	**Estimate**	**Standard error**	***p*-value**
**Hokkaido Prefecture**				
Beef cattle farm	59.1 (54.6–63.5)	Reference	0.095	-
Dairy farm	63.8 (57.4–69.7)	0.196	0.136	0.150
Pig farm	77.1 (71.5–81.9)	0.846	0.151	<0.001
Layer farm	67.2 (57.5–75.7)	0.350	0.212	0.100
Broiler farm (entire Japan)	88.9 (85.4–91.7)^*^	1.714	0.160^*^	<0.001
**Saitama Prefecture**				
Beef cattle farm	57.6 (54.9–60.3)	Reference	0.056	-
Dairy farm	58.5 (55.2–61.7)	0.036	0.069	0.601
Pig farm	60.0 (55.8–64.0)	0.098	0.088	0.264
Layer farm	64.9 (60.7–68.8)	0.306	0.091	0.001
Broiler farm (entire Japan)	88.9 (87.1–90.5)^*^	1.776	0.088^*^	<0.001

* *Note that standard errors of the same dataset were estimated differently according to the models*.

When the SRHM compliance rates of the same livestock species were compared between the two prefectures, only pig farms showed a significant difference (Hokkaido > Saitama, difference in logit = 0.811, standard error [SE] = 0.214, *p* < 0.001); no significant differences were seen for beef, dairy, or layer farms (*p* > 0.05).

**Figure 2:**
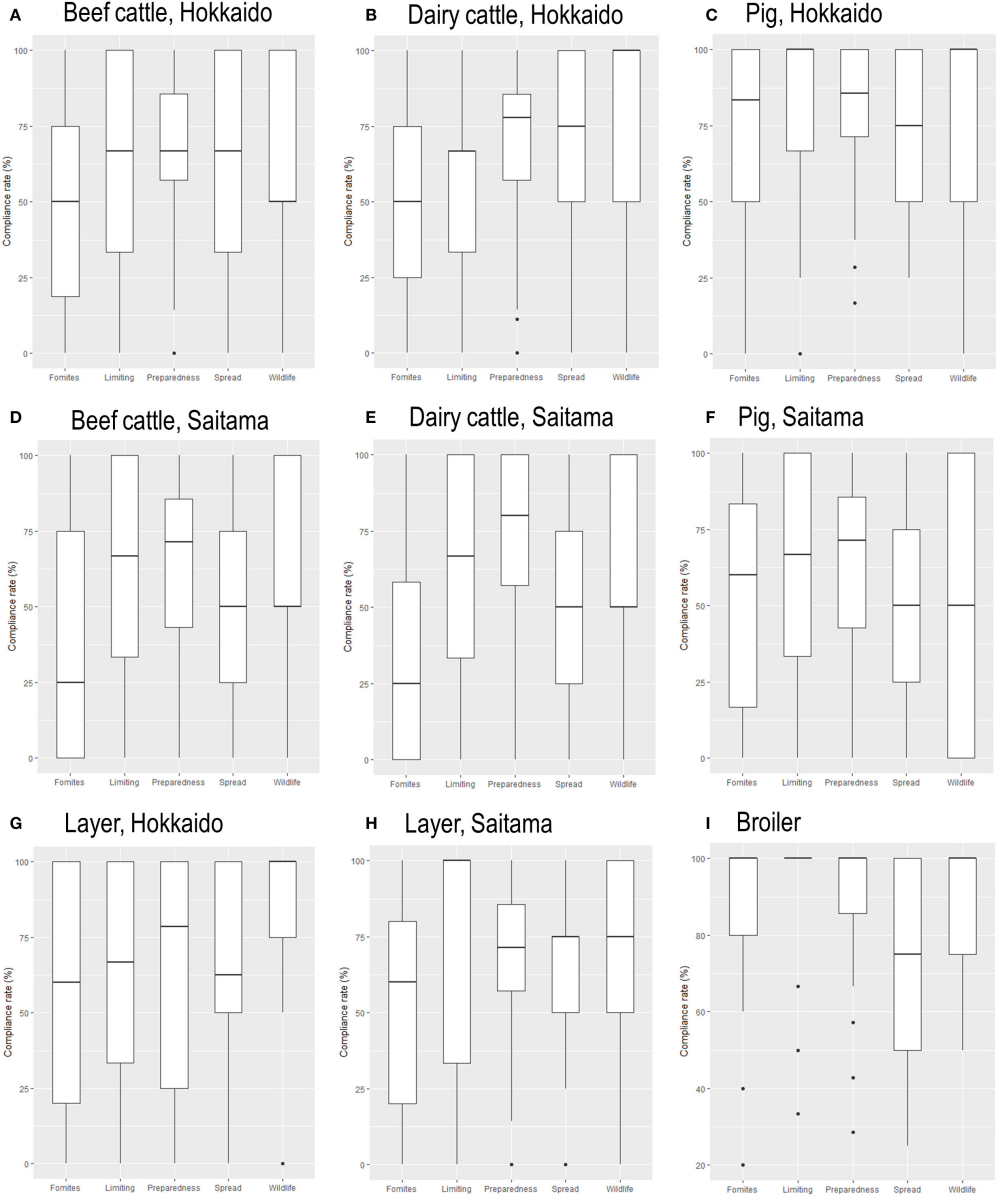
Box plots of category-specific Standard Rearing Hygiene Management compliance rates for **(A,D)** beef cattle, dairy **(B,E)**, pig **(C,F)**, and layer farms **(G,H)** in Hokkaido and Saitama Prefectures, and broiler farms **(I)**. Categories include the prevention of disease incursion from fomites and animals, limitation of access to a farm, maintenance of preparedness, prevention of within-farm spread, and prevention of incursion from wildlife, from left to right.

Figure 2 shows the category-specific SRHM compliance rates of the different livestock species in both prefectures and broiler. The beef cattle and dairy farms in both Hokkaido and Saitama Prefectures had particularly poor compliance with the SRHM items for the prevention of disease incursion from fomites compared with other categories. By contrast, the compliance rate associated with the SRHM category to maintain preparedness was high for both beef and dairy cattle farms in both prefectures. The SRHM categories to limit access and to prevent disease incursion with wildlife were particularly high for pig farms in Hokkaido Prefecture. A tendency toward poor prevention of within-farm disease spread was observed for pig farms in Saitama Prefecture. While the compliance rate associated with preventing disease incursion with wildlife was high in layer farms in Hokkaido, the other category specific compliance rates varied greatly in layer farms in both prefectures. For broiler farms, the compliance rate associated with the prevention of within-farm spread was lower than any other category. The item-specific compliance rates for beef cattle, dairy, pig, layer, and broiler farms are described in Supplementary Tables 1–5.

### Comparison of Farm Capacity and Density Between Hokkaido and Saitama Prefectures

Table 4 shows the results of comparisons of farm capacity and density between Hokkaido and Saitama Prefectures. For all livestock species, the farms in Hokkaido Prefecture were significantly larger than those in Saitama Prefecture in terms of the numbers of animals/birds. The numbers of workers were significantly larger in Hokkaido than in Saitama Prefecture for all livestock sectors except for in layer farms (*p* = 0.674). Farm owners were significantly younger in Hokkaido than in Saitama Prefecture for all livestock sectors except for layer farms (*p* = 0.235). The availability of a successor tended to be higher in beef cattle farms in Hokkaido than in Saitama Prefecture, but no difference was seen in the other sectors. Beef cattle and dairy farms were geographically closer to each other in Hokkaido than in Saitama Prefecture, but pig farms were more distant from each other in Hokkaido Prefecture.

**Table 4:** Comparison of livestock farm capacity and density between Hokkaido and Saitama Prefectures.

**Items**	**Hokkaido**	**Saitama**	**Statistics**	***p*-value**
**Beef Cattle Farm**				
Age of owner	48.5	63.6	*t* = −7.4, df = 139.2	<0.001
Number of animals (farrow-to-finisher)	161.4 (*n* = 44)	98.7 (*n* = 10)	W = 306	0.027
Number of animals (fattening)	64.0 (*n* = 2)	317.9 (*n* = 28)	-	-
Number of animals (growing)	44.3 (*n* = 4)	212.6 (*n* = 7)	W = 13	0.927
Number of animals (reproduction)	71.2 (*n* = 44)	20.6 (*n* = 21)	W = 813	<0.001
Total number of workers	3.5	2.9	Log = 0.18, se = 0.09	0.051
Availability of successor	61.8%	43.1%	*x*^2^ = 3.7, df = 1	0.055
Nearest distance to other beef farm	1.6 km	2.3 km	W = 1893.5	0.026
**Dairy Cattle Farm**				
Age of owner	53.4	61.5	W = 2967.5	<0.001
Number of animals	113.6	44.7	Log = 0.93, se = 0.02	<0.001
Total number of workers	3.3	2.6	Log = 0.22, se = 0.08	0.005
Availability of successor	37.0%	31.9%	*x*^2^ = 0.4, df = 1	0.528
Nearest distance to other dairy farm	1.4 km	2.1 km	W = 3747	0.026
**Pig Farm**				
Age of owner	55.8	60.0	W = 1055.5	0.028
Number of animals (farrow-to-finisher)	4,109.0 (*n* = 59)	1,024.5 (*n* = 41)	W = 1711.5	<0.001
Number of animals (fattening)	432.9 (*n* = 7)	774.3 (*n* = 3)	W = 17	0.628
Number of animals (reproduction)	62 (*n* = 1)	-	-	-
Total number of workers	6.4	3.0	Log = 0.77, se = 0.24	0.002
Availability of successor	46.2%	41.9%	*x*^2^ = 0.1, df = 1	0.809
Nearest distance to other pig farm	7.0 km	2.5 km	W = 1883.5	0.013
**Layer Farm**				
Age of owner	56.5	62.3	*t* = −1.24, df = 14.3	0.235
Number of animals	242,146.0	40,375.6	Log = 1.79, se = 0.59	0.004
Total number of workers	10.1	8.4	Log = 0.18, se = 0.41	0.674
Availability of successor	23.1%	45.7%	*x*^2^ = 1.2, df = 1	0.274
Nearest distance to other layer farm	8.4	4.1	W = 140	0.389

### Structural Equation Modeling (SEM)

**Figure 3:**
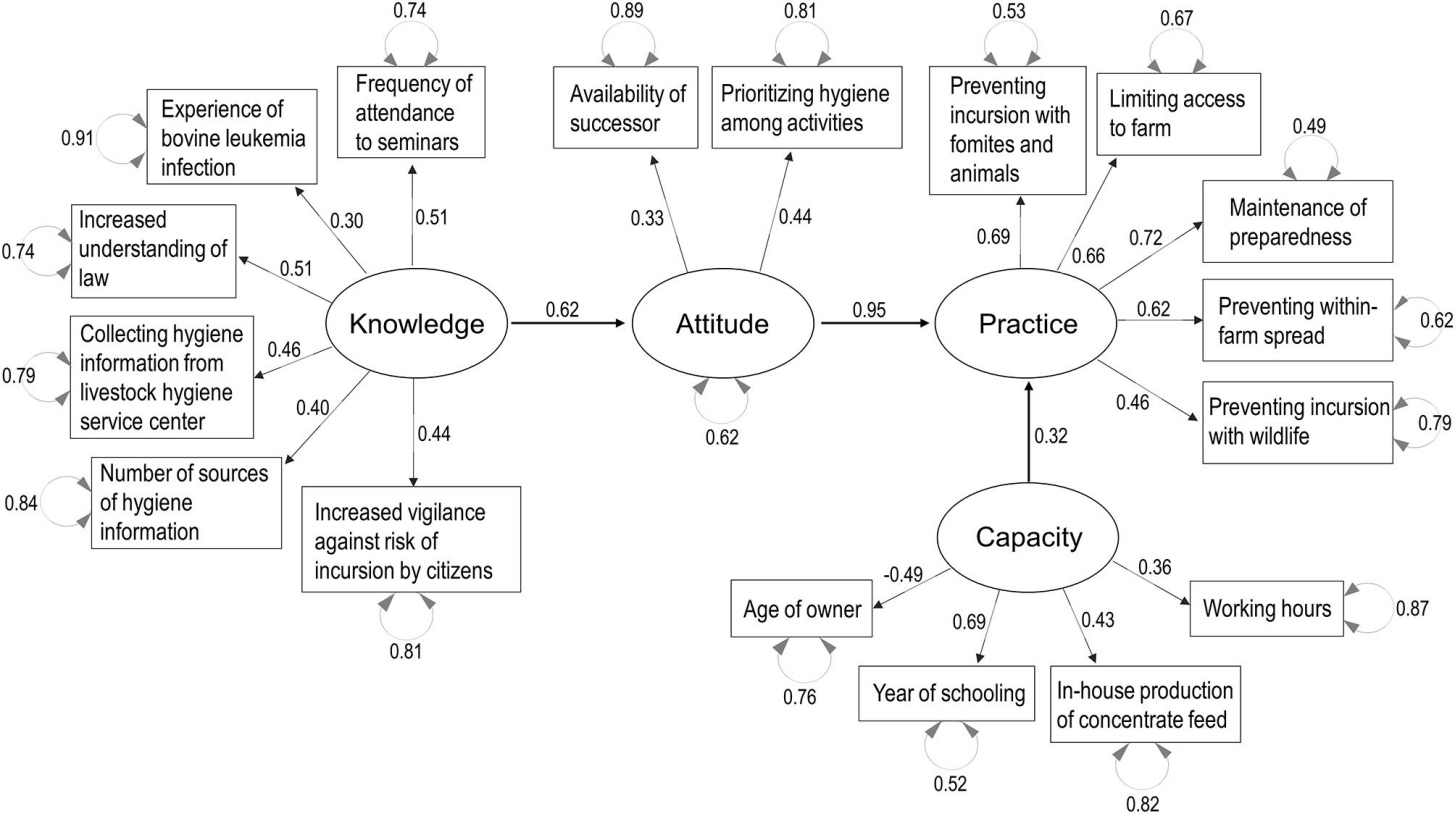
Structural equation modeling path graph for beef cattle farms. The ellipses and rectangles indicate latent and measured variables, respectively, and the values on the arrows are standardized factor loadings. The round arrows connected to latent and measured variables indicate disturbances.

**Figure 4:**
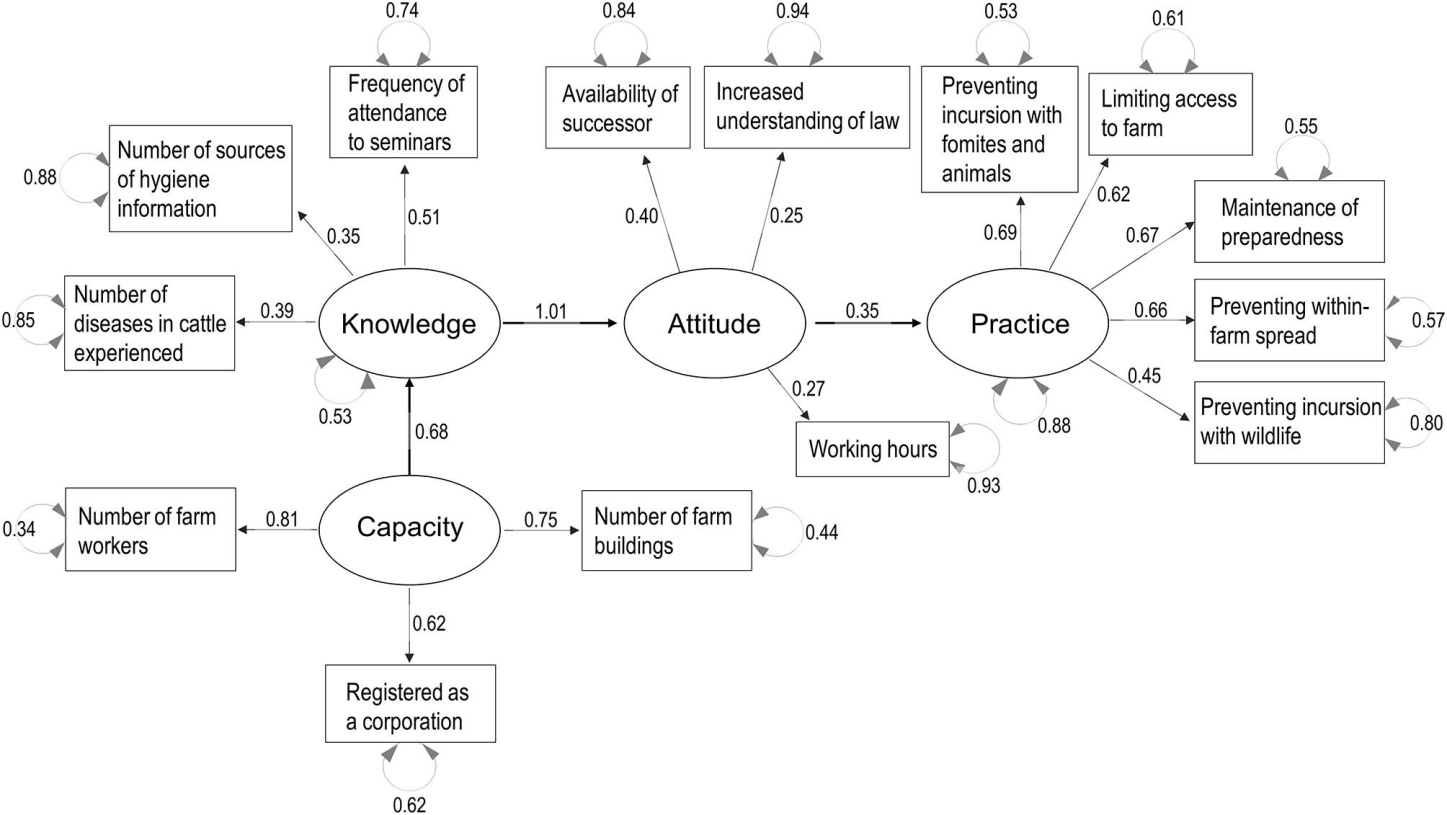
Structural equation modeling path graph for dairy farms. The ellipses and rectangles indicate latent and measured variables, respectively, and the values on the arrows are standardized factor loadings. The round arrows connected to latent and measured variables indicate disturbances.

**Figure 5:**
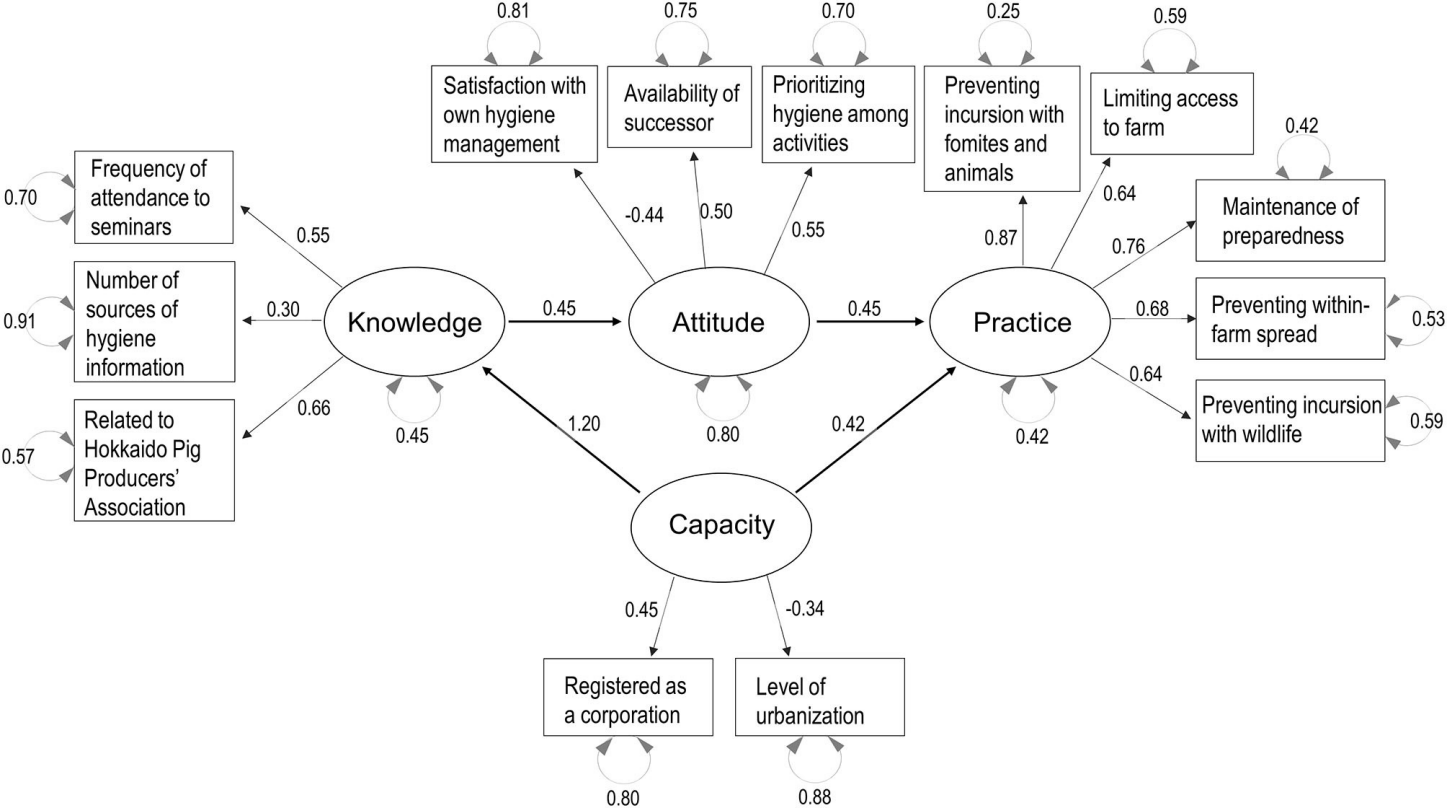
Structural equation modeling path graph for pig farms. The ellipses and rectangles indicate latent and measured variables, respectively, and the values on the arrows are standardized factor loadings. The round arrows connected to latent and measured variables indicate disturbances.

**Figure 6:**
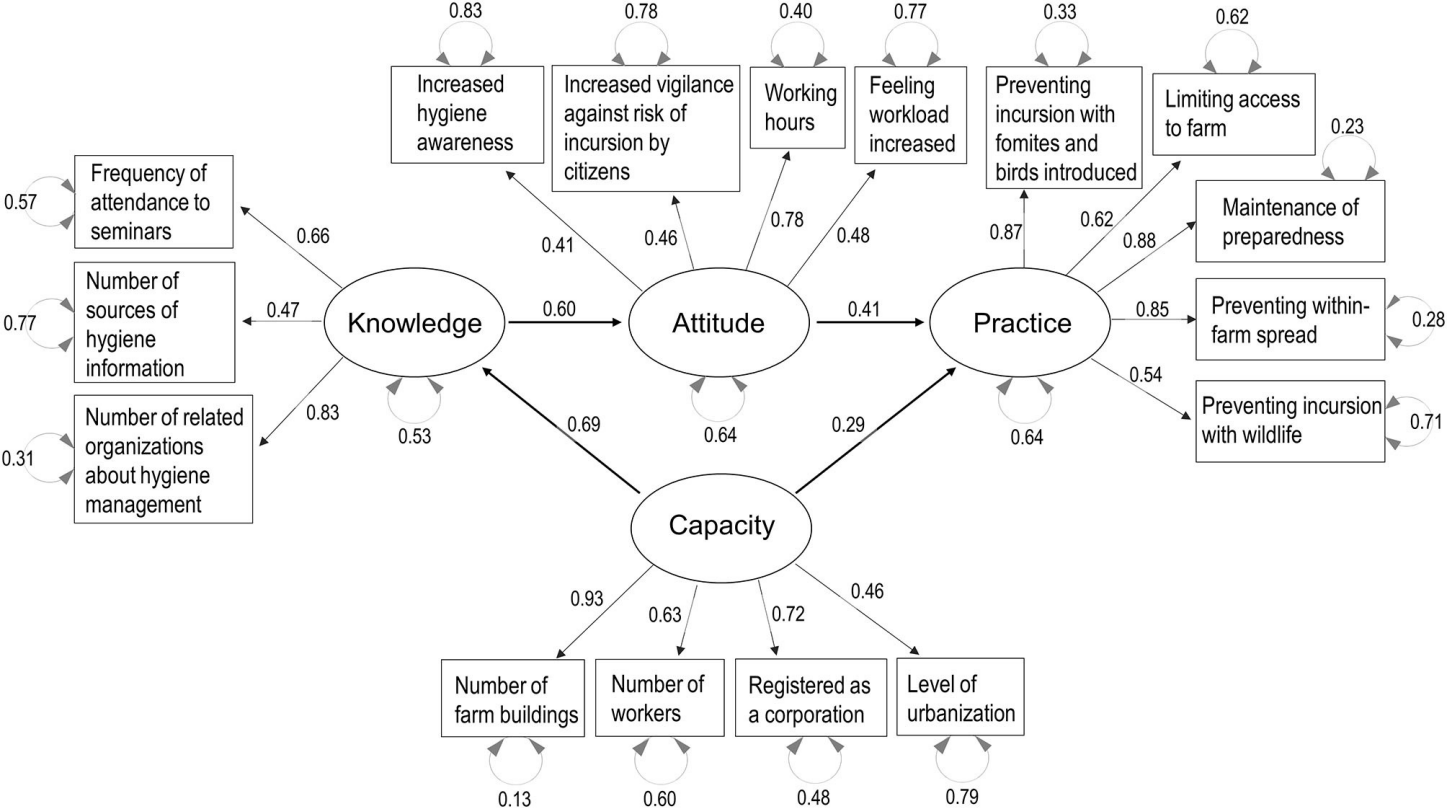
Structural equation modeling path graph for layer farms. The ellipses and rectangles indicate latent and measured variables, respectively, and the values on the arrows are standardized factor loadings. The round arrows connected to latent and measured variables indicate disturbances.

**Figure 7:**
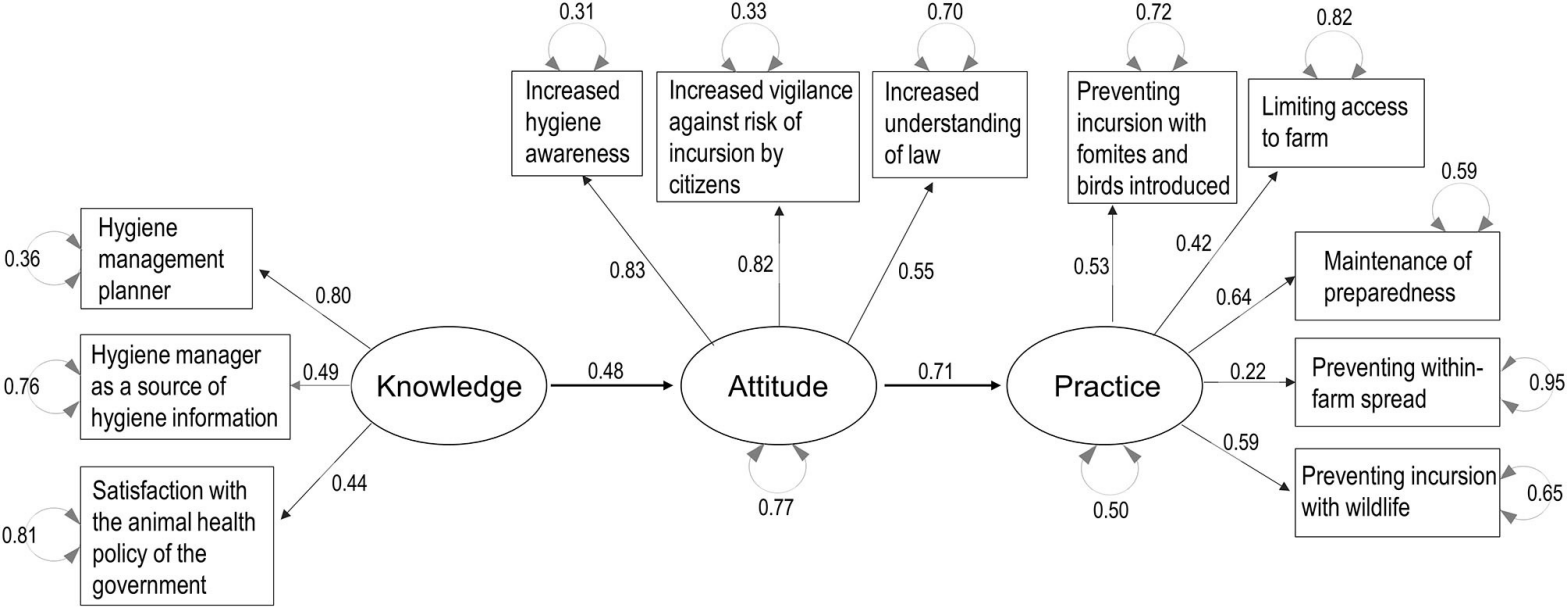
Structural equation modeling path graph for broiler farms. The ellipses and rectangles indicate latent and measured variables, respectively, and the values on the arrows are standardized factor loadings. The round arrows connected to latent and measured variables indicate disturbances.

Parallel analysis scree plots generally supported the number of factors (four factors) needed to consider the decision-making process structure, and the default SEM structure included the KAP-Capacity framework. Figures 3–7 show the SEM path graphs on the structures of the decision-making process to practice the SRHM items for beef, dairy, pig, layer, and broiler farms, respectively, and Supplementary Tables 6–10 show the detailed statistics. After excluding data with missing responses, 95, 192, 97, 36, and 84 responses from beef, dairy, pig, layer, and broiler farms were used in the SEM. The ellipses and rectangles in the figures indicate latent and measured variables, respectively. The measured variables connected to each latent variable were selected using exploratory factor analysis beforehand. The round arrows connecting to each of the latent and measured variables are disturbances, or in other words, variances. The values on the arrows in the figures show the SEM coefficients in tables, which are standardized factor loadings.

Regarding beef farms, flows in which better knowledge enhanced higher attitudes, and higher attitudes and greater capacity improved biosecurity practices, were significant (*p* < 0.05, Figure 3 and Supplementary Table 6, respectively). Knowledge was explained by learning behavior (source of information and attendance to seminars), disease experience, and increased understanding of law and vigilance after the revision of the SRHM. Attitude was explained by the availability of a successor and the prioritization of hygiene. Capacity was explained by a younger owner, longer working hours, higher education levels, and in-house production of concentrate feed. Compliance with all SRHM categories was significantly associated with practice (*p* < 0.05, Supplementary Table 6).

Regarding dairy farms, flows were significant in which knowledge was enhanced by greater capacity, better knowledge enhanced higher attitudes, and higher attitudes induced biosecurity practices (*p* < 0.001, Figure 4 and Supplementary Table 7). Capacity was explained by larger farm size (numbers of workers and buildings) and registration as a corporation, as compared with family-owned farms. Knowledge was explained by learning behavior (source of information and attendance to seminars) and disease experience. Attitude was explained by the availability of a successor, diligence (longer working hours), and increased understanding of the law after the revision of the SRHM. Practice was explained by compliance with all SRHM categories (*p* < 0.001, Supplementary Table 7).

Regarding pig farms, the decision-making process flow from knowledge and attitude to practice was similar to that for beef and dairy cattle farms, but capacity enhanced both knowledge and practice (Figure 5 and Supplementary Table 8). Knowledge was explained by learning behavior (source of information and attendance to seminars) and relation with the Hokkaido Pig Producers' Association. Attitude was explained by lower satisfaction with own hygiene management, availability of a successor, and prioritization of hygiene among activities. Capacity was explained by registration as a corporation as compared with a family-owned farm, and located in a rural as compared with a peri-urban area. Practice was again explained by compliance with all SRHM categories (*p* < 0.001, Supplementary Table 8).

Regarding layer farms, the decision-making process showed exactly the same structure as that for pig farms (Figure 6 and Supplementary Table 9). Knowledge was explained by learning behavior (source of information and attendance to seminars) and the number of related organizations regarding hygiene management. Attitude was explained by increased hygiene awareness and vigilance after the revision of the SRHM, as well as diligence (longer working hours and increased perceived workload). Capacity was explained by larger farm size (numbers of workers and buildings), registration as a corporation as compared with a family-owned farm, and located in rural area as compared with a peri-urban area. Practice was explained by compliance with all SRHM categories (*p* < 0.001, Supplementary Table 9).

Regarding broiler farms, the decision-making process flow from knowledge and attitude to practice was consistent with the other livestock sectors. However, capacity did not remain in the structure model (Figure 7 and Supplementary Table 10). Knowledge was explained by the hygiene manager being a source of hygiene information and satisfaction with the animal health policy of the Japanese government. The categorical variable selection of a hygiene management planner was included because the model did not pass the fitness criteria when it was excluded. Attitude was explained by increased hygiene awareness, vigilance, and understanding of the law after the revision of the SRHM. Practice was explained by the prevention of incursion from fomites, introduced animals, and wildlife, the limitation of access to a farm, and the maintenance of preparedness (*p* < 0.001); however, the prevention of within-farm spread was not a significant factor (coefficient = 0.22, SE = 0.13, *p* = 0.076, Supplementary Table 10).

All the final models passed the criteria for the chi-square *p*-value, TLI, RMSEA, and SRMR (Supplementary Tables 6–10).

## Discussion

This study aimed to provide a better understanding of the decision-making process in biosecurity practices on Japanese livestock farms in order to develop an extension program to improve biosecurity engagement and compliance. Although the response rates were acceptable for a postal survey, both the rates and the actual number of respondents varied between species and areas. The response rates were higher in Saitama Prefecture than Hokkaido Prefecture for all the species (beef cattle, dairy, pig, and layer farms), and this might be due to the closer relationship with the Saitama Livestock Association, which covered all the sectors. The number of farmers responding was the smallest in layer farms in Hokkaido Prefecture, and this reflected the difficulty of access without farmers' association.

Comparisons between livestock sectors found that the already highly commercialized broiler sector had tighter biosecurity than any other sector. In comparisons between Hokkaido and Saitama Prefectures, the SRHM compliance rate was significantly different only for pig farms (higher in Hokkaido Prefecture), which may be explained by factors such as capacity, including a younger owner and larger numbers of workers and animals on a farm in Hokkaido, or potentially more active engagement by the Hokkaido Pig Farmers' Association (as shown in the SEM results). Hokkaido Prefecture is characterized by intensive food production; among Japan's 47 prefectures, it has the largest numbers of beef and dairy cattle, the fifth largest number of pigs, and ninth largest number of layer birds, compared with Saitama Prefecture, which had the 29th, 21st, 21st, and 16th largest numbers, respectively, according to the 2013 livestock census (43); however, the compliance rates did not differ between the two prefectures in other sectors. Although the age of the owner and farm size were significantly different, the causality of the effect of socioeconomic factors on the difference in SRHM compliance rates between the two prefectures cannot be discussed further based on only these comparisons.

Comparisons between SRHM categories in each livestock sector suggested differences in biosecurity strategies between sectors. In beef cattle and dairy farms, while the preparedness for a disease outbreak was well-maintained, practices to prevent disease incursion from fomites and introduced animals, such as through the disinfection of vehicles, hands, and shoes of visitors, and the quarantining of introduced animals, were the most poorly conducted. Beef and dairy farmers complained about a lack of land and facilities to segregate animals for the quarantine period during FGDs. However, beef and dairy farmers should be aware that disease introduction can result in a greater economic burden than preparing an adequate quarantine facility. Intensive swine farms in Hokkaido made substantial efforts to prevent disease incursion from fomites and animals through biosecurity practices such as limiting access to a farm. This may be because farmers are aware of the apparent economic losses that can be incurred due to viral infectious diseases such as PED and porcine reproductive respiratory syndrome (44). Conversely, compliance with the SRHM category of preventing within-farm spread was weak, even for the intensive pig farms in Hokkaido Prefecture, which elucidated the challenges remaining in securing adequate space to reduce animal density and conduct all-in–all-out practices in intensive production systems. Layer farms in both prefectures did not have particularly high or low SRHM compliance categories, except preventing disease incursion from wildlife in Hokkaido, and this might have been due to their smaller sample size compared with other sectors. In Japan, the avian influenza vaccine is not used for layer hens, and thus, outbreaks of HPAI have been occurring in layer farms (14). As shown in Figure 2, the high compliance rate for the category of preventing incursion with wildlife in intensified layer farms in Hokkaido Prefecture was comparable to that of broiler farms. For broiler farms, systematic biosecurity strategies to prevent disease incursion from outside the farm are well-observed, but similar to pig farms, challenges remain in preventing within-farm spread, probably because of the intensity of the farming system.

The results of the SEM in all sectors studied suggest that greater knowledge of farm hygiene enhances positive attitudes toward hygiene, which determines the conduct of hygiene practice. In reality, attitude in itself may influence both knowledge uptake and behavior. However, our model assured the significant flow from knowledge *via* attitudes to practice. The KAP model has been applied in many countries for a wide variety of health and animal health problems (32, 45); however, during our FGDs, several farmers in different sectors described that even though they wanted to, they could not tighten biosecurity because farm labor and facilities are limited. We conceptualized that capacity, which is equivalent to self-efficacy in PMT (23) and PBC in the TPB (28), can be a prerequisite to high biosecurity performance. Although we hypothesized that knowledge, attitude, and capacity would be required to conduct biosecurity practices, these four components were not purposively selected in our statistical analyses. Parallel analysis scree plots indicated that four factors could generally explain the variance and covariance for livestock farming, and exploratory factor analyses identified the measured variables in the questionnaires to explain these factors; therefore, the KAP-Capacity decision-making structure was plausible, even statistically. Observation of the latent variable, capacity was useful in understanding the characteristics of the industries. The SEM results for the pig and layer farms suggested that in the default biosecurity decision-making structure, capacity is a driving force to increase the knowledge and feasibility needed to conduct the practices. A structure containing the flow from attitude to capacity, meaning that high motivation can increase capacity, was also tested (not indicated in the results), but the model fit was unacceptable, indicating that the SEM model cannot explain such a long-term effect of motivation on capacity development. For broiler farms, capacity did not remain in the model, probably because all the broiler producing companies studied had sufficient capacity to maintain high biosecurity. For dairy farms, even with sufficient capacity, the conduct of biosecurity measures may depend on better attitudes toward hygiene, as there was no significant linkage between capacity and practice. However, capacity enhanced knowledge, which would lead to better attitudes toward hygiene, and strengthening capacity would contribute to the higher SRHM compliance. Conversely for beef farms, limited capacity may be a physical obstacle to conduct these practices. Interestingly, the exchangeability of measured variables explaining different latent variables was observed between livestock sectors. For example, increased understanding of the law and vigilance after the revision of the SRHM explained knowledge in beef cattle farms, and attitude in other sectors, which suggests that knowledge, attitude, and even capacity are related and an experience or a response may constitute a personality that determines the strength of the KAP-Capacity process flow.

When planning policies to upgrade farm biosecurity, the provision of knowledge through networking with prefectural livestock hygiene centers, and of hygiene training by these centers, was identified as the most important step. The cost (46) and effectiveness (47) of biosecurity measures influence the motivation of farmers, and the provision of such evidence-based information should increase attitudes toward the SRHM. Policies to strengthen capacity, such as intensification through a shift to a corporate farm and support for smallholder farms, can also be effective. The standardized factor loads in the SEM models are useful for planning detailed intervention strategies and developing monitoring schemes for knowledge, attitude, and capacity; the variables with factor loads distant from zero have a strong relationship with the latent variables, and may be important factors for interventions and monitoring. However, the factor loads can be used as a guidance when developing monitoring schemes, but not as a direct manual or checklist, as each production system and biosecurity program must be addressed individually with practical knowledge. In addition, decision-making process frameworks for protective practices in health and animal health, such as the HBM, PMT, TRA, and TPB, have already described detailed qualitative factors, and thus, it is advisable that veterinary officers at both the national and local government levels understand these theories when carrying out detailed planning for hygiene guidance.

This study had three limitations: (1) the representativeness of farmers, (2) the use of self-administered questionnaires, and (3) the use of binary responses in the analyses. Regarding (1), due to budget constraints and the expected time required for coordination, postal surveys other than for broiler farms were conducted in only two prefectures. Among pig and beef cattle farms, there are reproduction farms, fattening farms, and farrow-to-finisher farms; however, due to the limited numbers of farmers who were reached to participate in this study, all farm types were analyzed together. In Japan, there are small numbers of sheep and goat farmers and those for indigenous breeds of chicken for eggs and meats, and these farmers were excluded from the present study. This study was initially designed to conduct statistics using GLMs, so a sample size calculation for the SEM was not conducted. However, the SEM models had adequate degrees of freedom and passed the model fit criteria, and thus, the results can be considered reliable. Regarding (2), our study used self-administered questionnaires and the results may be affected by information bias. Regarding (3), the default estimation method in SEM, maximum likelihood, assumes multivariate normality in between-variable relationships, and normal distributions of these variables (34). Therefore, in the SEM analyses, the WLSM was used. However, future studies should collect data using a Likert-type scale rather than binary responses. In addition, the postal surveys were conducted between 2013 and 2014, and the SRHM compliance rates in 2018, enumerated by the veterinarians at the prefectural livestock hygiene centers, were much higher (48).

## Conclusions

The study results support the hypothesis that the decision-making process for conducting farm biosecurity starts from the acquisition of good hygiene knowledge, which enhances attitudes, and better attitudes are a trigger to conduct enhanced biosecurity practices. Capacity is an important factor to improve both hygiene knowledge and biosecurity practice. Intensification is a key factor for achieving tighter biosecurity, but well-designed facilities and management plans are needed to ensure the prevention of within-farm disease spread. SEM is a potentially powerful tool for collecting data to support the design of effective and well-targeted intervention programs to improve farm biosecurity.

## Data Availability Statement

The raw data supporting the conclusions of this article will be made available by the authors, without undue reservation.

## Ethics Statement

Ethical review and approval was not required for the study on human participants in accordance with the local legislation and institutional requirements. The patients/participants provided their written informed consent to participate in this study.

## Author Contributions

KM planned, coordinated stakeholders, conducted fieldworks, analyses, supervised, and wrote the manuscript. ES and LH conducted analyses including SEM and wrote the manuscript. SH conducted univariable and multivariable analyses on poultry sectors. YN, YT, TW, and SA conducted fieldworks and analyses. HK conducted and supervised univariable and multivariable analyses. All authors contributed to the article and approved the submitted version.

## Conflict of Interest

The authors declare that the research was conducted in the absence of any commercial or financial relationships that could be construed as a potential conflict of interest. The handling editor declared a past co-authorship with one of the authors KM.

## References

[1] European Food Safety Authority Scientific opinion on African swine fever. EFSA J. (2010) 8:1556 10.2903/j.efsa.2010.1556

[2] Guinat C Gogin A Blome S Keil G Pollin R Pfeiffer DU. Transmission routes of African swine fever virus to domestic pigs: current knowledge and future research directions. Vet Rec. (2016) 178:262–7. 10.1136/vr.103593 26966305 PMC4819659

[3] Pikalo J Schoder ME Sehl J Breithaupt A Tignon M Cay AB. The African swine fever virus isolate Belgium 2018/1 shows high virulence in European wild boar. Transbound Emerg Dis. (2020) 67:1654–9. 10.1111/tbed.13503 32009303

[4] Zhou X Li N Luo Y Liu YE Miao F Chen T. Emergence of African swine fever in China. Transbound Emerg Dis. (2018) 65:1482–4. 10.1111/tbed.12989 30102848

[5] Food and Agriculture Organization ASF Situation in Asia Update. (2020). Available online at: http://www.fao.org/ag/againfo/programmes/en/empres/ASF/situation_update.html (accessed April 2, 2020).

[6] Ito S Jurado C Sánchez-Vizcaíno JM Isoda N. Quantitative risk assessment of African swine fever virus introduction to Japan via pork products brought in air passengers' luggage. Transbound Emerg Dis. (2020) 67:894–905. 10.1111/tbed.13414 31692238

[7] Peck DF Foot and mouth outbreak: lessons for mental health services. Adv Psychiatr Treat. (2005) 11:270–6. 10.1192/apt.11.4.270

[8] Hibi J Kurosawa A Watanabe T Kadowaki H Watari M. Post-traumatic stress disorder in participants of foot-and-mouth disease epidemic control in Miyazaki, Japan, in 2010. J Vet Med Sci. (2015) 77:953–9. 10.1292/jvms.14-0512 25843114 PMC4565818

[9] Kadowaki H Kayano T Tobinaga T Tsutsumi A Watari M Makita K. Analysis of factors associated with hesitation to restart farming after depopulation of animals due to 2010 foot-and-mouth disease epidemic in Japan. J Vet Med Sci. (2016) 78:1251–9. 10.1292/jvms.15-0559 27149890 PMC5053925

[10] Toma L Stott A Heffernan C Ringrose S Gunn GJ. Determinants of biosecurity behaviour of British cattle and sheep farmers—a behavioural economics analysis. Prev Vet Med. (2013) 108:321–33. 10.1016/j.prevetmed.2012.11.009 23194894

[11] Ministry of Agriculture, Forestry, Fisheries of Japan Act on Domestic Animal Infectious Diseases Control. (2012). Available online at: http://www.maff.go.jp/e/policies/ap_health/animal/attach/pdf/index-19.pdf (accessed March 10, 2020).

[12] Ministry of Agriculture, Forestry, Fisheries of Japan Revisions of the Standards of Rearing Hygiene Management. (2016). Available online at: https://www.maff.go.jp/j/council/seisaku/eisei/goudou_02/attach/pdf/index-14.pdf (accessed March 10, 2020).

[13] Ministry of Agriculture, Forestry, Fisheries of Japan FY2010 Annual Report on Food, Agriculture and Rural Areas in Japan. (2011). Available online at: https://www.maff.go.jp/e/annual_report/2010/pdf/e_all.pdf (accessed March 5, 2020).

[14] Kishita Y Outbreaks and control measures of notifiable avian influenza in Japan. J Vet Epidemiol. (2016) 20:S11–3. 10.2743/jve.20.S11

[15] Ministry of Agriculture, Forestry, Fisheries of Japan Standard Rearing Hygiene Management. (2011). Available online at: https://www.maff.go.jp/j/syouan/douei/katiku_yobo/k_shiyou/attach/pdf/kako-12.pdf (accessed March 10, 2020).

[16] Toyomaki H Sekiguchi S Sasaki Y Sueyoshi M Makita K. Factors associated with farm-level infection of porcine epidemic diarrhea during the early phase of the epidemic in Japan in 2013 and 2014. Prev Vet Med. (2018) 150:77–85. 10.1016/j.prevetmed.2017.12.008 29406087

[17] Ministry of Agriculture, Forestry, Fisheries of Japan Standard Rearing Hygiene Management. (2017). Available online at: https://www.maff.go.jp/j/syouan/douei/katiku_yobo/k_shiyou/attach/pdf/index-2.pdf (accessed March 10, 2020).

[18] Ministry of Agriculture, Forestry, Fisheries of Japan The Situation of Hygienic Management in Livestock Rearing in 2018. (2018). Available online at: https://www.maff.go.jp/j/syouan/douei/katiku_yobo/k_shiyou/attach/pdf/index-49.pdf (accessed March 10, 2020).

[19] Isoda N Baba K Ito S Ito M Sakoda Y Makita K. Dynamics of classical swine fever spread in wild boar in 2018–2019, Japan. Pathogens. (2020) 9:119. 10.3390/pathogens9020119 32069897 PMC7169391

[20] Hayama Y Shimizu Y Murato Y Sawai K Yamamoto T. Estimation of infection risk on pig farms in infected wild boar areas—epidemiological analysis for the reemergence of classical swine fever in Japan in 2018. Prev Vet Med. (2020) 175:104873. 10.1016/j.prevetmed.2019.104873 31896501

[21] Rosenstock IM The health belief model and preventive health behavior. Health Educ. Monogr. (1974) 2:354–86. 10.1177/109019817400200405

[22] Janz NK Becker MH. The Health Belief Model: a decade later. Health Educ Q. (1984) 11:1–47. 10.1177/109019818401100101 6392204

[23] Prentice-Dunn S Rogers RW Protection motivation theory and preventive health: beyond the health belief model. Health Educ Res. (1986) 1:153–61. 10.1093/her/1.3.153

[24] Schemann K Taylor MR Toribio JALML Dhand NK. Horse owners' biosecurity practices following the first equine influenza outbreak in Australia. Prev Vet Med. (2011) 102:304–14. 10.1016/j.prevetmed.2011.08.002 21893356

[25] Schemann K Firestone SM Taylor MR Toribio JALML Ward MP Dhand NK. Perceptions of vulnerability to a future outbreak: a study of horse managers affected by the first Australian equine influenza outbreak. BMC Vet Res. (2013) 9:152. 10.1186/1746-6148-9-152 23902718 PMC3737030

[26] Fishbain M Ajzen I Belief, Attitude, Intention, and Behaviour: An Introduction to Theory and Research. Reading, MA: Addison-Wesley (1975).

[27] Alarcon P Wieland B Mateus ALP Dewberry C. Pig farmers' perceptions, attitudes, influences and management of information in the decision-making process for disease control. Prev Vet Med. (2014) 116:223–42. 10.1016/j.prevetmed.2013.08.004 24016600

[28] Ajzen I. The theory of planned behavior. Organ Behav Hum Decis Process. (1991) 50:179–211. 10.1016/0749-5978(91)90020-T21929476

[29] Garforth CJ Bailey AP Tranter RB. Farmers' attitudes to disease risk management in England: a comparative analysis of sheep and pig farmers. Prev Vet Med. (2013) 110:456–66. 10.1016/j.prevetmed.2013.02.018 23490144

[30] Launiala A How much can a KAP survey tell us about people's knowledge, attitudes and practices? Some observations from medical anthropology research on malaria in pregnancy in Malawi. Anthropol Matters. (2009) 11:1–13. 10.22582/am.v11i1.31

[31] Sambo M Lembo T Cleaveland S Ferguson HM Sikana L Simon C. Knowledge, attitudes and practices (KAP) about rabies prevention and control: a community survey in Tanzania. PLoS Negl Trop Dis. (2014) 8:e3310. 10.1371/journal.pntd.0003310 25473834 PMC4256472

[32] Kadowaki H Duc PP Sato K Phuong PTM Hagiwara K Makita K. Socio-economic factors associated with voluntary rabies control measures in Vietnam. Prev Vet Med. (2018) 157:105–14. 10.1016/j.prevetmed.2018.06.006 30086838

[33] Mueller R Basic Principles of Structural Equation Modeling. New York, NY: Springer-Verlag (1996). 10.1007/978-1-4612-3974-1

[34] Kline RB Principles and Practice of Structural Equation Modeling. 4th ed New York, NY, London: The Guilford Press (2016).

[35] Watanabe T Nakahara Y Asakura S Makita K Sociological factors influencing the practice and attitude towards pig farm hygiene in Hokkaido Prefecture, Japan. J Vet Epidemiol. (2015) 19:100–7. 10.2743/jve.19.100

[36] Japan Chicken Association A Member List of Japan Chicken Association in 2014. Tokyo (2014).

[37] Revelle W Procedures for Psychological, Psychometric, and Personality, Package “psych” Version 1.9.12.31. (2019). Available online at: https://cran.r-project.org/web/packages/psych/psych.pdf (accessed December 3, 2019).

[38] Bernaads CA Jennrich RI Gradient projection algorythms and software for arbitrary rotation criteria in factor analysis. Educ Psychol Meas. (2005) 65:676–96. 10.1177/0013164404272507

[39] Rosseel Y lavaan: an R package for structural equation modeling. J Stat Softw. (2012) 48:1–36. 10.18637/jss.v048.i02

[40] Hu LT Bentler PM Cutoff criteria for fit indexes in covariance structure analysis: conventional criteria versus new alternatives. Struct Equ Modeling. (1999) 6:1–55. 10.1080/10705519909540118

[41] Kline RB Global Fit Testing, Principles and Practice of Structural Equation Modeling. 4th ed New York, NY: Guilford Press (2005).

[42] R Core Team R: A Language and Environment for Statistical Computing. Vienna: R Foundation for Statistical Computing (2019). Available online at: https://www.R-project.org/ (accessed April 12, 2019).

[43] Ministry of Agriculture, Forestry, Fisheries of Japan Raiser Households of Livestock or Chickens, Livestock or Chickens Raised, Production of Raw Milk and Hen Eggs by Prefecture. Statistical Survey on Livestock (2013).

[44] Iseki H Morozumi T Takagi M Kawashima K Shibahara T Uenishi H. Genomic sequence and virulence evaluation of MN184A-like porcine reproductive and respiratory syndrome virus in Japan. Microbiol Immunol. (2016) 60:824–34. 10.1111/1348-0421.12455 27925288

[45] Cloete A Gerstenberg C Mayet N Tempia S. Brucellosis knowledge, attitudes and practices of a South African communal cattle keeper group. Onderstepoort J Vet. (2019) 86:1671. 10.4102/ojvr.v86i1.1671 30843408 PMC6407466

[46] Fraser RW Williams NT Powell LF Cook AJ. Reducing *Campylobacter* and *Salmonella* infection: two studies of the economic cost and attitude to adoption of on-farm biosecurity measures. Zoonoses Public Health. (2010) 57:109–15. 10.1111/j.1863-2378.2009.01295.x 19968845

[47] Gunn GJ Heffernan C Hall M McLeod A Hovi M. Measuring and comparing constraints to improved biosecurity amongst GB farmers, veterinarians and the auxiliary industries. Prev Vet Med. (2008) 84:310–23. 10.1016/j.prevetmed.2007.12.003 18282623

[48] Ministry of Agriculture, Forestry, Fisheries of Japan The Situation of Compliance to Standard Rearing Hygiene Management in Japan as of 2018 February 1st. (2018). Available online at: https://www.maff.go.jp/j/syouan/douei/katiku_yobo/k_shiyou/attach/pdf/index-87.pdf (accessed March 10, 2020).

