# Supplementary material for: Quantitative Understanding of the Decision-Making Process for Farm Biosecurity Among Japanese Livestock Farmers Using the KAP-Capacity Framework
Source: Front Vet Sci. 2020 Sep 11;7:614. doi: 10.3389/fvets.2020.00614 (PMC7517466; doi:10.3389/fvets.2020.00614)
Supplement: Supplementary file 1 [file Table_1.DOCX]

**Supplementary Table 1. Compliance rates for Standards of Rearing Hygiene Management (SRHM) items in beef cattle farms in Hokkaido and Saitama prefectures**

|  | Hokkaido |  | Saitama |  |
| --- | --- | --- | --- | --- |
| SRHM items | Complied/  response | Percentage | Complied/  response | Percentage |
| ***Preventing incursion with fomites and animals*** |  |  |  |  |
| Disinfection of vehicles | 38/83 | 45.8% | 24/64 | 37.5% |
| Disinfection of hands and shoes of those who enter to the farm building | 50/89 | 56.2% | 28/65 | 43.1% |
| Cleaning or disinfection of materials directly used for animals when carry them in hygiene control area | 40/86 | 46.5% | 27/64 | 42.2% |
| Prohibition of carrying clothes and shoes used abroad into the farm | 22/79 | 27.8% | 28/61 | 45.9% |
| Quarantine of animals under segregation from other animals for certain period when introducing into the farm | 48/82 | 58.5% | 25/63 | 39.7% |
| ***Limiting access to the farm*** |  |  |  |  |
| Segregation of hygiene control area from the other areas | 39/84 | 46.4% | 25/63 | 39.7% |
| Placement of a signboard indicating the hygiene control area | 65/90 | 72.2% | 48/65 | 73.8% |
| Limit of access for those who entered other farms or recently returned from abroad | 38/87 | 43.7% | 29/65 | 44.6% |
| ***Prevention of incursion from wildlife*** |  |  |  |  |
| Prevention of wildlife feces entering to feeding and water facilities | 29/85 | 34.1% | 27/62 | 43.5% |
| Provision of drinkable water for domestic animals | 77/88 | 87.5% | 58/65 | 89.2% |
| ***Prevention of within-farm spread*** |  |  |  |  |
| Change (disposal) or disinfection of materials to which body fluid of animals got attached, at each use | 39/83 | 47.0% | 22/62 | 35.5% |
| Cleaning and disinfection of a barn or cage after being emptied | 65/86 | 75.6% | 39/64 | 60.9% |
| Rearing animals with suitable density | 53/82 | 64.6% | 51/65 | 78.5% |
| ***Maintenance of preparedness*** |  |  |  |  |
| Collecting up-to-date information on prevention of animal infectious diseases | 49/85 | 57.6% | 23/62 | 37.1% |
| Immediate report of specific symptoms by law to the Livestock Hygiene Service Centre (LHSC) and restriction of animal movement | 43/79 | 54.4% | 38/64 | 59.4% |
| Immediate call of veterinarians when animals are sick without specific symptoms by law | 65/82 | 79.3% | 50/65 | 76.9% |
| Daily health check of animals | 86/88 | 97.7% | 60/65 | 92.3% |
| Removal of dirt and health check at selling out animals | 81/88 | 92.0% | 60/65 | 92.3% |
| Securing a land to bury culled animals | 34/80 | 42.5% | 38/64 | 59.4% |
| Record keeping for early identification of source of infection | 36/81 | 44.4% | 27/63 | 42.9% |
