# Supplementary material for: Quantitative Understanding of the Decision-Making Process for Farm Biosecurity Among Japanese Livestock Farmers Using the KAP-Capacity Framework
Source: Front Vet Sci. 2020 Sep 11;7:614. doi: 10.3389/fvets.2020.00614 (PMC7517466; doi:10.3389/fvets.2020.00614)
Supplement: Supplementary file 2 [file Table_2.DOCX]

**Supplementary Table 2. Compliance rates for Standards of Rearing Hygiene Management (SRHM) items in dairy farms in Hokkaido and Saitama prefectures**

|  | Hokkaido |  | Saitama |  |
| --- | --- | --- | --- | --- |
| SRHM items | Complied/  response | Percentage | Complied/  response | Percentage |
| ***Preventing incursion with fomites and animals*** |  |  |  |  |
| Disinfection of vehicles | 31/82 | 37.8% | 25/130 | 19.2% |
| Disinfection of hands and shoes of those who enter to the farm building | 54/83 | 65.0% | 65/132 | 49.2% |
| Cleaning or disinfection of materials directly used for animals when carry them in hygiene control area | 41/81 | 50.6% | 54/129 | 41.9% |
| Prohibition of carrying clothes and shoes used abroad into the farm | 27/80 | 33.8% | 45/123 | 36.6% |
| Quarantine of animals under segregation from other animals for certain period when introducing into the farm | 26/82 | 31.7% | 43/127 | 33.9% |
| ***Limiting access to the farm*** |  |  |  |  |
| Segregation of hygiene control area from the other areas | 34/84 | 40.5% | 63/127 | 49.6% |
| Placement of a signboard indicating the hygiene control area | 63/84 | 75.0% | 93/131 | 71.0% |
| Limit of access for those who entered other farms or recently returned from abroad | 41/83 | 49.4% | 56/133 | 42.1% |
| ***Prevention of incursion from wildlife*** |  |  |  |  |
| Prevention of wildlife feces entering to feeding and water facilities | 40/82 | 48.8% | 61/130 | 46.9% |
| Provision of drinkable water for domestic animals | 78/85 | 91.8% | 114/133 | 85.7% |
| ***Prevention of within-farm spread*** |  |  |  |  |
| Change (disposal) or disinfection of materials to which body fluid of animals got attached, at each use | 50/82 | 61.0% | 62/131 | 47.3% |
| Cleaning and disinfection of a barn or cage after being emptied | 77/85 | 90.6% | 93/133 | 69.9% |
| Rearing animals with suitable density | 62/83 | 74.7% | 90/130 | 69.2% |
| ***Maintenance of preparedness*** |  |  |  |  |
| Collecting up-to-date information on prevention of animal infectious diseases | 51/81 | 63.0% | 67/128 | 52.3% |
| Immediate report of specific symptoms by law to the Livestock Hygiene Service Centre (LHSC) and restriction of animal movement | 66/83 | 79.5% | 87/132 | 65.9% |
| Immediate call of veterinarians when animals are sick without specific symptoms by law | 76/85 | 89.4% | 111/132 | 84.1% |
| Daily health check of animals | 83/85 | 97.6% | 126/133 | 94.7% |
| Removal of dirt and health check at selling out animals | 65/85 | 76.5% | 105/133 | 78.9% |
| Securing a land to bury culled animals | 41/84 | 48.8% | 89/131 | 67.9% |
| Record keeping for early identification of source of infection | 54/85 | 63.5% | 75/129 | 58.1% |
