# Supplementary material for: Quantitative Understanding of the Decision-Making Process for Farm Biosecurity Among Japanese Livestock Farmers Using the KAP-Capacity Framework
Source: Front Vet Sci. 2020 Sep 11;7:614. doi: 10.3389/fvets.2020.00614 (PMC7517466; doi:10.3389/fvets.2020.00614)
Supplement: Supplementary file 3 [file Table_3.DOCX]

**Supplementary Table 3. Compliance rates for Standards of Rearing Hygiene Management (SRHM) items in pig farms in Hokkaido and Saitama prefectures**

|  | Hokkaido |  | Saitama |  |
| --- | --- | --- | --- | --- |
| SRHM items | Complied/  response | Percentage | Complied/  response | Percentage |
| ***Preventing incursion with fomites and animals*** |  |  |  |  |
| Disinfection of vehicles | 43/67 | 64.2% | 27/43 | 62.8% |
| Disinfection of hands and shoes of those who enter to the farm building | 52/67 | 77.6% | 28/44 | 63.6% |
| Provision of clothes and shoes only for  hygiene control area | 45/67 | 67.2% | 25/44 | 56.8% |
| Cleaning or disinfection of materials directly used for animals when carry them in hygiene control area | 53/67 | 79.1% | 22/40 | 55.0% |
| Prohibition of carrying clothes and shoes used abroad into the farm | 48/63 | 76.2% | 19/36 | 48.7% |
| Quarantine of animals under segregation from other animals for certain period when introducing into the farm | 43/66 | 65.2% | 22/41 | 53.7% |
| Heat treatment of recycled feed | 46/62 | 74.2% | 15/35 | 42.9% |
| ***Limiting access to the farm*** |  |  |  |  |
| Segregation of hygiene control area from the other areas | 52/67 | 77.6% | 25/44 | 56.8% |
| Placement of a signboard indicating the hygiene control area | 53/67 | 79.1% | 29/43 | 67.4% |
| Limit of access for those who entered other farms or recently returned from abroad | 56/67 | 83.5% | 27/43 | 62.8% |
| ***Prevention of incursion from wildlife*** |  |  |  |  |
| Prevention of wildlife feces entering to feeding and water facilities | 49/66 | 74.2% | 21/43 | 48.8% |
| Provision of drinkable water for domestic animals | 61/66 | 92.4% | 31/42 | 73.8% |
| ***Prevention of within-farm spread*** |  |  |  |  |
| Change (disposal) or disinfection of materials to which body fluid of animals got attached, at each use | 29/66 | 43.9% | 9/39 | 23.1% |
| Cleaning and disinfection of a barn or cage after being emptied | 60/66 | 91.0% | 28/43 | 65.1% |
| Rearing animals with suitable density | 46/65 | 70.8% | 24/42 | 57.1% |
| ***Maintenance of preparedness*** |  |  |  |  |
| Collecting up-to-date information on prevention of animal infectious diseases | 49/65 | 75.4% | 28/43 | 65.1% |
| Immediate report of specific symptoms by law to the Livestock Hygiene Service Centre (LHSC) and restriction of animal movement | 55/66 | 83.3% | 25/42 | 59.5% |
| Immediate call of veterinarians when animals are sick without specific symptoms by law | 49/65 | 75.4% | 25/42 | 59.5% |
| Daily health check of animals | 65/67 | 97.0% | 39/43 | 90.7% |
| Removal of dirt and health check at selling out animals | 62/67 | 92.5% | 39/43 | 90.7% |
| Securing a land to bury culled animals | 54/67 | 80.6% | 27/43 | 62.8% |
| Record keeping for early identification of source of infection | 42/66 | 63.6% | 18/43 | 41.9% |
