# Supplementary material for: Quantitative Understanding of the Decision-Making Process for Farm Biosecurity Among Japanese Livestock Farmers Using the KAP-Capacity Framework
Source: Front Vet Sci. 2020 Sep 11;7:614. doi: 10.3389/fvets.2020.00614 (PMC7517466; doi:10.3389/fvets.2020.00614)
Supplement: Supplementary file 4 [file Table_4.DOCX]

**Supplementary Table 4. Compliance rates for Standards of Rearing Hygiene Management (SRHM) items in layer farms in Hokkaido and Saitama prefectures.**

|  | Hokkaido |  | Saitama |  |
| --- | --- | --- | --- | --- |
| SRHM items | Complied/  response | Percentage | Complied/  response | Percentage |
| ***Preventing incursion with fomites and animals*** |  |  |  |  |
| Disinfection of vehicles | 9/20 | 45.0% | 17/38 | 44.7% |
| Disinfection of hands and shoes of those who enter to the farm building | 12/20 | 60.0% | 28/39 | 71.8% |
| Provision of clothes and shoes only for hygiene control area | 10/19 | 52.6% | 21/39 | 53.8% |
| Cleaning or disinfection of materials directly used for animals when carry them in hygiene control area | 12/19 | 63.2% | 19/38 | 50.0% |
| Prohibition of carrying clothes and shoes used abroad into the farm | 11/19 | 57.9% | 16/32 | 50.0% |
| Quarantine of animals under segregation from other animals for certain period when introducing into the farm | 12/20 | 60.0% | 19/36 | 52.8% |
| ***Limiting access to the farm*** |  |  |  |  |
| Segregation of hygiene control area from the other areas | 13/20 | 65.0% | 22/38 | 57.9% |
| Placement of a signboard indicating the hygiene control area | 11/20 | 55.0% | 30/39 | 76.9% |
| Limit of access for those who entered other farms or recently returned from abroad | 15/20 | 75.0% | 30/38 | 78.9% |
| ***Prevention of incursion from wildlife*** |  |  |  |  |
| Prevention of wildlife feces entering to feeding and water facilities | 17/19 | 89.5% | 30/39 | 76.9% |
| Provision of drinkable water for layer hens | 11/19 | 57.9% | 9/36 | 25.0% |
| Placement of nets preventing entrance of wild birds | 19/20 | 95.0% | 36/39 | 92.3% |
| Pest control, repair of damaged roof and walls | 19/20 | 95.0% | 30/39 | 76.9% |
| ***Prevention of within-farm spread*** |  |  |  |  |
| Change (disposal) or disinfection of materials to which body fluid of animals got attached, at each use | 8/19 | 42.1% | 6/32 | 18.8% |
| Cleaning and disinfection of a barn or cage after being emptied | 15/20 | 75.0% | 34/38 | 89.5% |
| Rearing animals with suitable density | 17/19 | 89.5% | 34/39 | 87.2% |
| ***Maintenance of preparedness*** |  |  |  |  |
| Collecting up-to-date information on prevention of animal infectious diseases | 12/19 | 63.2% | 24/38 | 63.2% |
| Immediate report of specific symptoms by law to the Livestock Hygiene Service Centre (LHSC) and restriction of animal movement | 13/19 | 68.4% | 21/36 | 58.3% |
| Immediate call of veterinarians when animals are sick without specific symptoms by law | 12/19 | 63.2% | 14/36 | 38.9% |
| Daily health check of animals | 16/20 | 80.0% | 38/39 | 97.4% |
| Removal of dirt and health check at selling out animals | 13/20 | 65.0% | 28/36 | 77.8% |
| Securing a land to bury culled animals | 12/20 | 60.0% | 28/37 | 75.7% |
| Record keeping for early identification of source of infection | 8/19 | 42.1% | 16/33 | 48.5% |
