# Supplementary material for: Quantitative Understanding of the Decision-Making Process for Farm Biosecurity Among Japanese Livestock Farmers Using the KAP-Capacity Framework
Source: Front Vet Sci. 2020 Sep 11;7:614. doi: 10.3389/fvets.2020.00614 (PMC7517466; doi:10.3389/fvets.2020.00614)
Supplement: Supplementary file 5 [file Table_5.DOCX]

**Supplementary Table 5. Compliance rates for Standards of Rearing Hygiene Management (SRHM) items in broiler farms in Japan.**

| SRHM items | Complied/  response | Percentage |
| --- | --- | --- |
| ***Preventing incursion with fomites and animals*** |  |  |
| Disinfection of vehicles | 85/95 | 89.5% |
| Disinfection of hands and shoes of those who enter to the farm building | 90/94 | 95.7% |
| Provision of clothes and shoes only for hygiene control area | 87/92 | 94.6% |
| Cleaning or disinfection of materials directly used for animals when carry them in hygiene control area | 89/93 | 95.7% |
| Prohibition of carrying clothes and shoes used abroad into the farm | 74/88 | 84.1% |
| Quarantine of animals under segregation from other animals for certain period when introducing into the farm | 64/91 | 70.3% |
| ***Limiting access to the farm*** |  |  |
| Segregation of hygiene control area from the other areas | 88/95 | 92.6% |
| Placement of a signboard indicating the hygiene control area | 93/94 | 98.9% |
| Limit of access for those who entered other farms or recently returned from abroad | 89/93 | 95.7% |
| ***Prevention of incursion from wildlife*** |  |  |
| Prevention of wildlife feces entering to feeding and water facilities | 90/93 | 96.8% |
| Provision of drinkable water for broilers | 74/94 | 78.7% |
| Placement of nets preventing entrance of wild birds | 89/92 | 96.7% |
| Pest control, repair of damaged roof and walls | 88/92 | 95.7% |
| ***Prevention of within-farm spread*** |  |  |
| Change (disposal) or disinfection of materials to which body fluid of animals got attached, at each use | 48/92 | 52.2% |
| Cleaning and disinfection of a barn or cage after being emptied | 92/94 | 97.9% |
| Rearing animals with suitable density | 29/93 | 31.2% |
| ***Maintenance of preparedness*** |  |  |
| Collecting up-to-date information on prevention of animal infectious diseases | 78/92 | 84.8% |
| Immediate report of specific symptoms by law to the Livestock Hygiene Service Centre (LHSC) and restriction of animal movement | 92/94 | 97.9% |
| Immediate call of veterinarians when animals are sick without specific symptoms by law | 85/93 | 91.4% |
| Daily health check of animals | 93/94 | 98.9% |
| Removal of dirt and health check at selling out animals | 87/94 | 92.6% |
| Securing a land to bury culled animals | 73/93 | 78.5% |
| Record keeping for early identification of source of infection | 80/94 | 85.1% |
