# Supplementary material for: Quantitative Understanding of the Decision-Making Process for Farm Biosecurity Among Japanese Livestock Farmers Using the KAP-Capacity Framework
Source: Front Vet Sci. 2020 Sep 11;7:614. doi: 10.3389/fvets.2020.00614 (PMC7517466; doi:10.3389/fvets.2020.00614)
Supplement: Supplementary file 6 [file Table_6.DOCX]

**Supplementary table 6. Measurement and regression results regarding structural equation modelling for beef cattle farms**

| Variable | Coefficient | SE | p-value |
| --- | --- | --- | --- |
| ***Structure*** |  |  |  |
| **Knowledge** to **Attitude** | 0.62 | 0.15 | <0.001 |
| **Attitude** to **Practice** | 0.95 | 0.18 | <0.001 |
| **Capacity** to **Practice** | 0.32 | 0.13 | 0.012 |
| ***Regression*** |  |  |  |
| **Knowledge** to |  |  |  |
| Frequency of attendance to seminars | 0.51 | 0.11 | <0.001 |
| Experience of bovine leukemia infection | 0.30 | 0.13 | 0.016 |
| Increased understanding of law after the revision of SRHM | 0.51 | 0.12 | <0.001 |
| Collecting hygiene information from livestock hygiene service  center | 0.46 | 0.10 | <0.001 |
| Number of sources of hygiene information | 0.40 | 0.11 | <0.001 |
| Increased vigilance against risk of incursion from citizens | 0.44 | 0.12 | <0.001 |
| **Attitude** to |  |  |  |
| Availability of successor | 0.33 | 0.11 | 0.003 |
| Prioritizing hygiene management among farming activities | 0.44 | 0.12 | <0.001 |
| **Practice** to |  |  |  |
| Preventing incursion with fomites and animals introduced | 0.69 | 0.07 | <0.001 |
| Limiting access to farm | 0.66 | 0.07 | <0.001 |
| Maintenance of preparedness | 0.72 | 0.07 | <0.001 |
| Preventing within-farm spread | 0.62 | 0.08 | <0.001 |
| Preventing incursion with wildlife | 0.46 | 0.10 | <0.001 |
| **Capacity** to |  |  |  |
| Age of owner | -0.49 | 0.14 | <0.001 |
| Year of schooling | 0.69 | 0.17 | <0.001 |
| In-house production of concentrate feed | 0.43 | 0.17 | 0.014 |
| Working hours | 0.36 | 0.11 | 0.001 |
| ***Fit measures*** |  |  |  |
| Number of observation used | 95 |  |  |
| Degrees of freedom | 115 |  |  |
| *X*^2^ *p*-value | 0.887 |  |  |
| Tucker-Lewis Index | 1.053 |  |  |
| Root Mean Square Error of Approximation | 0.000 |  |  |
| Standardized Root Mean Square Error of Approximation | 0.082 |  |  |
