# Supplementary material for: Quantitative Understanding of the Decision-Making Process for Farm Biosecurity Among Japanese Livestock Farmers Using the KAP-Capacity Framework
Source: Front Vet Sci. 2020 Sep 11;7:614. doi: 10.3389/fvets.2020.00614 (PMC7517466; doi:10.3389/fvets.2020.00614)
Supplement: Supplementary file 7 [file Table_7.DOCX]

**Supplementary Table 7. Measurement and regression results regarding structural equation modelling for dairy cattle farms**

| Variable | Coefficient | SE | p-value |
| --- | --- | --- | --- |
| ***Structure*** |  |  |  |
| **Knowledge** to **Attitude** | 1.01 | 0.18 | <0.001 |
| **Attitude** to **Practice** | 0.35 | 0.11 | <0.001 |
| **Capacity** to **Knowledge** | 0.68 | 0.10 | <0.001 |
| ***Regression*** |  |  |  |
| **Knowledge** to |  |  |  |
| Frequency of attendance to seminars | 0.51 | 0.10 | <0.001 |
| Number of sources of hygiene information | 0.35 | 0.11 | 0.002 |
| Number of diseases in cattle experienced | 0.39 | 0.07 | <0.001 |
| **Attitude** to |  |  |  |
| Availability of successor | 0.40 | 0.10 | <0.001 |
| Increased understanding of law after the revision of SRHM | 0.25 | 0.10 | 0.011 |
| Working hours | 0.27 | 0.08 | <0.001 |
| **Practice** to |  |  |  |
| Preventing incursion with fomites | 0.69 | 0.05 | <0.001 |
| Limiting access to farm | 0.62 | 0.06 | <0.001 |
| Maintenance of preparedness | 0.67 | 0.06 | <0.001 |
| Preventing within-farm spread | 0.66 | 0.06 | <0.001 |
| Preventing incursion with wildlife | 0.45 | 0.07 | <0.001 |
| **Capacity** to |  |  |  |
| Registered as a corporation | 0.62 | 0.11 | <0.001 |
| Number of farm workers | 0.81 | 0.08 | <0.001 |
| Number of farm buildings | 0.75 | 0.11 | <0.001 |
| ***Fit measures*** |  |  |  |
| Number of observation used | 192 |  |  |
| Degrees of freedom | 74 |  |  |
| *X*^2^ *p*-value | 0.695 |  |  |
| Tucker-Lewis Index | 1.017 |  |  |
| Root Mean Square Error of Approximation | 0.000 |  |  |
| Standardized Root Mean Square Error of Approximation | 0.061 |  |  |
