# Supplementary material for: Quantitative Understanding of the Decision-Making Process for Farm Biosecurity Among Japanese Livestock Farmers Using the KAP-Capacity Framework
Source: Front Vet Sci. 2020 Sep 11;7:614. doi: 10.3389/fvets.2020.00614 (PMC7517466; doi:10.3389/fvets.2020.00614)
Supplement: Supplementary file 8 [file Table_8.DOCX]

**Supplementary Table 8. Measurement and regression results regarding structural equation modelling for pig farms**

| Variable | Coefficient | SE | p-value |
| --- | --- | --- | --- |
| ***Structure*** |  |  |  |
| **Knowledge** to **Attitude** | 0.45 | 0.17 | 0.009 |
| **Attitude** to **Practice** | 0.45 | 0.16 | 0.006 |
| **Capacity** to **Knowledge** | 1.20 | 0.20 | <0.001 |
| **Capacity** to **Practice** | 0.42 | 0.13 | 0.001 |
| ***Regression*** |  |  |  |
| **Knowledge** to |  |  |  |
| Frequency of attendance to seminars | 0.55 | 0.07 | <0.001 |
| Number of sources of hygiene information | 0.30 | 0.11 | 0.007 |
| Related to Hokkaido Pig Producers’ Association | 0.66 | 0.08 | <0.001 |
| **Attitude** to |  |  |  |
| Availability of successor | 0.50 | 0.12 | <0.001 |
| Satisfaction with own hygiene management | -0.44 | 0.11 | <0.001 |
| Prioritizing hygiene among farm activities | 0.55 | 0.12 | <0.001 |
| **Practice** to |  |  |  |
| Preventing incursion with fomites | 0.87 | 0.04 | <0.001 |
| Limiting access to farm | 0.64 | 0.07 | <0.001 |
| Maintenance of preparedness | 0.76 | 0.06 | <0.001 |
| Preventing within-farm spread | 0.68 | 0.06 | <0.001 |
| Preventing incursion with wildlife | 0.64 | 0.07 | <0.001 |
| **Capacity** to |  |  |  |
| Registered as a corporation | 0.45 | 0.12 | <0.001 |
| Level of urbanization | -0.34 | 0.11 | 0.001 |
| ***Fit measures*** |  |  |  |
| Number of observation used | 97 |  |  |
| Degrees of freedom | 61 |  |  |
| *X*^2^ *p*-value | 0.989 |  |  |
| Tucker-Lewis Index | 1.053 |  |  |
| Root Mean Square Error of Approximation | 0.000 |  |  |
| Standardized Root Mean Square Error of Approximation | 0.065 |  |  |
