# Supplementary material for: Quantitative Understanding of the Decision-Making Process for Farm Biosecurity Among Japanese Livestock Farmers Using the KAP-Capacity Framework
Source: Front Vet Sci. 2020 Sep 11;7:614. doi: 10.3389/fvets.2020.00614 (PMC7517466; doi:10.3389/fvets.2020.00614)
Supplement: Supplementary file 9 [file Table_9.DOCX]

**Supplementary Table 9. Measurement and regression results regarding structural equation modelling for layer farms**

| Variable | Coefficient | SE | p-value |
| --- | --- | --- | --- |
| ***Structure*** |  |  |  |
| **Knowledge** to **Attitude** | 0.60 | 0.19 | 0.001 |
| **Attitude** to **Practice** | 0.41 | 0.13 | 0.001 |
| **Capacity** to **Knowledge** | 0.69 | 0.15 | <0.001 |
| **Capacity** to **Practice** | 0.29 | 0.14 | 0.034 |
| ***Regression*** |  |  |  |
| **Knowledge** to |  |  |  |
| Frequency of attendance to seminars | 0.66 | 0.17 | <0.001 |
| Number of sources of hygiene information | 0.47 | 0.20 | 0.017 |
| Number of related organizations about hygiene management | 0.83 | 0.12 | <0.001 |
| **Attitude** to |  |  |  |
| Increased hygiene awareness after the revision of SRHM | 0.41 | 0.17 | 0.017 |
| Increased vigilance against risk of incursion by citizens after the revision of SRHM | 0.46 | 0.15 | 0.002 |
| Working hours | 0.78 | 0.16 | <0.001 |
| Feeling that workload was increased after the revision of SRHM | 0.48 | 0.20 | 0.017 |
| **Practice** to |  |  |  |
| Preventing incursion with fomites | 0.82 | 0.07 | <0.001 |
| Limiting access to farm | 0.62 | 0.18 | <0.001 |
| Maintenance of preparedness | 0.88 | 0.06 | <0.001 |
| Preventing within-farm spread | 0.85 | 0.11 | <0.001 |
| Preventing incursion with wildlife | 0.54 | 0.16 | <0.001 |
| **Capacity** to |  |  |  |
| Number of farm buildings | 0.93 | 0.08 | <0.001 |
| Number of workers | 0.63 | 0.09 | <0.001 |
| Registered as a corporation | 0.72 | 0.13 | <0.001 |
| Level of urbanization | 0.46 | 0.12 | 0.001 |
| ***Fit measures*** |  |  |  |
| Number of observation used | 36 |  |  |
| Degrees of freedom | 100 |  |  |
| *X*^2^ *p*-value | 1.00 |  |  |
| Tucker-Lewis Index | 1.180 |  |  |
| Root Mean Square Error of Approximation | 0.000 |  |  |
| Standardized Root Mean Square Error of Approximation | 0.100 |  |  |
