# Supplementary material for: Quantitative Understanding of the Decision-Making Process for Farm Biosecurity Among Japanese Livestock Farmers Using the KAP-Capacity Framework
Source: Front Vet Sci. 2020 Sep 11;7:614. doi: 10.3389/fvets.2020.00614 (PMC7517466; doi:10.3389/fvets.2020.00614)
Supplement: Supplementary file 10 [file Table_10.DOCX]

**Supplementary Table 10. Measurement and regression results regarding structural equation modelling for broiler farms**

| Variable | Coefficient | SE | p-value |
| --- | --- | --- | --- |
| ***Structure*** |  |  |  |
| **Knowledge** to **Attitude** | 0.48 | 0.14 | <0.001 |
| **Attitude** to **Practice** | 0.71 | 0.10 | <0.001 |
| ***Regression*** |  |  |  |
| **Knowledge** to |  |  |  |
| Hygiene management planner (farm manager, hygiene manager, or others) | 0.80 | 0.11 | <0.001 |
| Hygiene manager is the source of hygiene information | 0.49 | 0.11 | <0.001 |
| Satisfaction with the animal health policy of the government | 0.44 | 0.15 | 0.003 |
| **Attitude** to |  |  |  |
| Increased hygiene awareness after the revision of SRHM | 0.83 | 0.05 | <0.001 |
| Increased vigilance against risk of incursion by citizens after the revision of SRHM | 0.82 | 0.04 | <0.001 |
| Increased understanding of law after the revision of SRHM | 0.55 | 0.07 | <0.001 |
| **Practice** to |  |  |  |
| Preventing incursion with fomites | 0.53 | 0.12 | <0.001 |
| Limiting access to farm | 0.42 | 0.13 | <0.001 |
| Maintenance of preparedness | 0.64 | 0.12 | <0.001 |
| Preventing within-farm spread | 0.22 | 0.13 | 0.076 |
| Preventing incursion with wildlife | 0.59 | 0.12 | <0.001 |
| ***Fit measures*** |  |  |  |
| Number of observation used | 84 |  |  |
| Degrees of freedom | 42 |  |  |
| *X*^2^ *p*-value | 0.296 |  |  |
| Tucker-Lewis Index | 0.983 |  |  |
| Root Mean Square Error of Approximation | 0.036 |  |  |
| Standardized Root Mean Square Error of Approximation | 0.098 |  |  |
